# Efficient Internet-of-Things Cyberattack Depletion Using Blockchain-Enabled Software-Defined Networking and 6G Network Technology

**DOI:** 10.3390/s23249690

**Published:** 2023-12-07

**Authors:** Abdul Razaque, Joon Yoo, Gulnara Bektemyssova, Majid Alshammari, Tolganay T. Chinibayeva, Saule Amanzholova, Aziz Alotaibi, Dauren Umutkulov

**Affiliations:** 1 School of Computing, Gachon University, Seongnam 13120, Republic of Korea; joon.yoo@gachon.ac.kr; 2 Department of Computer Engineering and Information System, International Information Technology University, Almaty 050000, Kazakhstan; t.temirbolatova@iitu.edu.kz (T.T.C.); d.umutkulov@iitu.edu.kz (D.U.); 3 Computers and Information Technology College, Taif University, Taif 26571, Saudi Arabia; m.alshammari@tu.edu.sa; 4 Department of Cybersecurity, International Information Technology University, Almaty 050000, Kazakhstan; s.amanzholova@iitu.edu.kz

**Keywords:** IoT, 6G technology, cyberattack, blockchain technology, software-defined networking, virtual network function, edge computing, consensus blockchain technology

## Abstract

Low-speed internet can negatively impact incident response by causing delayed detection, ineffective response, poor collaboration, inaccurate analysis, and increased risk. Slow internet speeds can delay the receipt and analysis of data, making it difficult for security teams to access the relevant information and take action, leading to a fragmented and inadequate response. All of these factors can increase the risk of data breaches and other security incidents and their impact on IoT-enabled communication. This study combines virtual network function (VNF) technology with software -defined networking (SDN) called virtual network function software-defined networking (VNFSDN). The adoption of the VNFSDN approach has the potential to enhance network security and efficiency while reducing the risk of cyberattacks. This approach supports IoT devices that can analyze large volumes of data in real time. The proposed VNFSDN can dynamically adapt to changing security requirements and network conditions for IoT devices. VNFSDN uses threat filtration and threat-capturing and decision-driven algorithms to minimize cyber risks for IoT devices and enhance network performance. Additionally, the integrity of IoT devices is safeguarded by addressing the three risk categories of data manipulation, insertion, and deletion. Furthermore, the prioritized delegated proof of stake (PDPoS) consensus variant is integrated with VNFSDN to combat attacks. This variant addresses the scalability issue of blockchain technology by providing a safe and adaptable environment for IoT devices that can quickly be scaled up and down to pull together the changing demands of the organization, allowing IoT devices to efficiently utilize resources. The PDPoS variant provides flexibility to IoT devices to proactively respond to potential security threats, preventing or mitigating the impact of cyberattacks. The proposed VNFSDN dynamically adapts to the changing security requirements and network conditions, improving network resiliency and enabling proactive threat detection. Finally, we compare the proposed VNFSDN to existing state-of-the-art approaches. According to the results, the proposed VNFSDN has a 0.08 ms minimum response time, a 2% packet loss rate, 99.5% network availability, a 99.36% threat detection rate, and a 99.77% detection accuracy with 1% malicious nodes.

## 1. Introduction

Slow internet speeds during an incident response time can be caused by obsolete technology, network congestion, and a large number of network-connected devices [1]. This can result in slow download and upload speeds, high latency, and poor network stability, which can significantly impact the incident response [2]. Slow internet can lead to delayed response times, missed opportunities, customer dissatisfaction, and decreased productivity for the IoT devices [3]. Several solutions are available to address the issue of slow internet speeds in incident response. One solution is to use software-defined networking (SDN), which can improve the network efficiency, flexibility, and scalability for the IoT devices [4]. SDN allows for the centralized management of network resources, which can lead to a more efficient use of the available bandwidth [5]. Additionally, upgrading hardware and software and optimizing network configurations are effective ways to enhance network performance [6]. Another promising solution to improve internet speed is the use of virtual network functions (VNFs), which can be deployed on virtual machines to optimize the network infrastructure and enhance the overall network performance [7]. VNFs enable organizations to scale and manage their network resources effectively, allocate resources more efficiently, and improve network performance for IoT devices [8]. Internet speed is crucial in incident responses; however, a slow internet connection can result in sluggish reaction times, missed opportunities, unhappy clients, and diminished productivity. The causes of slow internet connections include outdated hardware, network congestion, and a large number of devices connected to a network [9]. Improving incident response times requires finding practical solutions for sluggish internet speeds. It is anticipated that the deployment of 6G covers the development of many new technologies and improves internet connection. Furthermore, 6G will perform a revolutionary role for new technologies such as smart surfaces, zero-energy IoT devices, advanced AI techniques, AI-powered automated devices, potential quantum computing systems, humanoid robots, AI-driven air interfaces, and self-sustained networks. Moreover, future trends of digital societies, such as massive AI and self-sustained networks, will also benefit from 6G [10]. Therefore, 6G is attractive to numerous applications, including UAV-based mobility, smart Grid 2.0, connected autonomous vehicles (CAV), hyper-intelligent healthcare, collaborative robots, Industry 5.0, Digital Twin, and Extended Reality. These applications might support many stakeholders and call for various levels of 6G security requirements. The security requirements and problems in 6G may vary greatly due to the novelty of these application domains and the potent adversaries. A federated-learning supported intrusion detection system (FSIDS) makes use of 6G-enabling technologies such software -defined networking, mobile edge computing, and network function virtualization [11]. DeepVulSeeker is a completely automated vulnerability identification platform that uses both code graph structures and semantic elements to find vulnerabilities. However, existing methods experience shortcomings [12].

To overcome this issue, the VNFSDN was proposed, which consists of two main components: VNF and SDN. The VNF is used to deploy intermediary devices over edge computing. The main function of an SDN is to manage intermediary devices centrally. The proposed VNFSDN is supported by 6G technology, which speeds up all processes. The VNF and SDN technologies are integrated for a faster response time for IoT devices.

Preemptive security rules are established in our proposed approach to prevent attacks. When the attacks are complete, the suggested approach, based on the outcome of the attack, supplements and enforces additional security rules. Additionally, it is challenging to establish ordinary packets and attacked packets. Thus, every packet passes via an SDN controller to apply an attack mitigation component that enables the server administrator to specify a flexible probability for the packet to be accommodated. The network-related functions listed in [13] were developed using a number of free source programs, which facilitates efficient communication.

**Figure 1:**
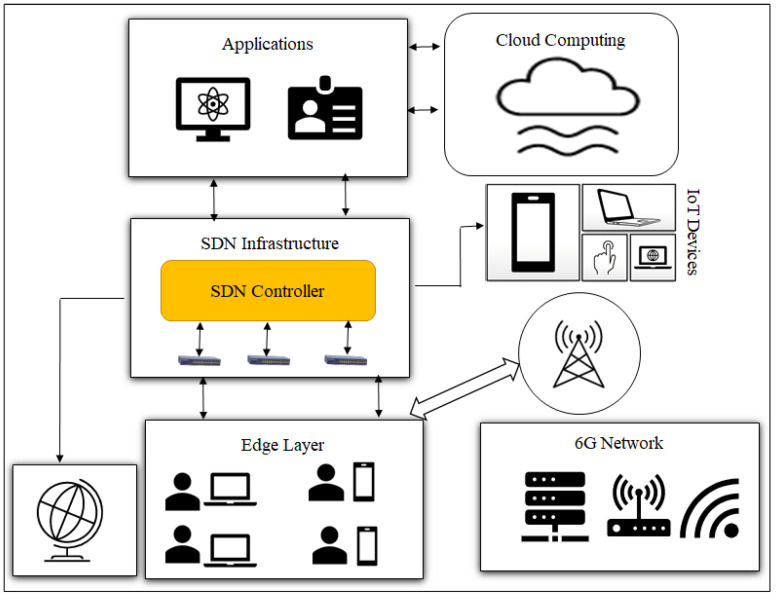
General concept of software-defined networking for IoT devices using edge computing and 6G technology.

The novel PDPoS algorithm is implemented to reduce the scalability of the blockchain technology to efficiently and securely combat against potential attacks for IoT devices. The proposed remedy presents a potential strategy for addressing the problem of sluggish internet speed and enhancing the general performance of the network for IoT devices. It may also enable improved security and centralized management. Figure 1 illustrates the general concept of software-defined networking for IoT devices using a 6G technology. Our proposed approach demonstrates how the integration of SDN and VNF technologies can create a flexible and scalable network infrastructure that can be easily managed and orchestrated.

### 1.1. Paper Organization

The remainder of the paper is organized as follows: Section 2 discusses the salient features of the existing methods in related work. Section 3 proposes an efficient IoT cyberattack depletion procedure based on VNFSDN and 6G. Furthermore, a threat filtration process for IoT devices is offered, as well as a threat collection and decision-driven process for IoT devices and modeling of a blockchain-enabled consensus algorithm (PDPoS) for cyberattack depletion. Section 4 presents the experimental setup and testing results. Section 5 provides a discussion of the results including the advantages of the proposed approach and its limitations. Finally, the conclusions and future work are summarized in Section 6.

### 1.2. Research Contributions

The contributions and novelty of this work can be summarized as follows:

- Virtual network function technology is used with software-defined networking to optimize network architecture and improve the overall network performance. IoT devices obtain faster response times, improved network security management, and high threat detection rates.
- VNFSDN incorporates threat filtration, collection, and decision-driven algorithms to avoid and reduce cyber risks for IoT devices and improve network performance. Furthermore, data integrity for IoT devices is protected by addressing three categories of risks (data deletion, data insertion, and data manipulation).
- Prioritized delegated proof of stake is an entirely novel consensus variant implemented to combat attacks. This variant addresses the scalability issue of blockchain technology by providing a safe and adaptable environment for IoT devices that can quickly be scaled up and down to suit the changing demands of the organization, allowing IoT devices to efficiently utilize resources.

### 1.3. Problem Identification and Significance

In today’s digital age, in which businesses and organizations rely heavily on the internet for their day-to-day operations, cyberattacks and slow internet speeds can have a significant impact on incident response times [14]. Slow internet connections can result from various factors, such as outdated hardware, network congestion, potential malicious cyberattacks, and the number of devices connected to the network [15]. The consequences of cyberattacks and slow internet speed can be severe, which lead to delayed response times, missed opportunities, customer dissatisfaction, and decreased productivity [16]. Incident response times and depletion of the cyberattack are critical for organizations, as they directly affect their ability to identify, contain, and mitigate security incidents. Cyberattacks and slow internet speeds can delay incident response times, allowing security breaches to persist for extended periods and leading to more significant damage to an organization’s reputation, finances, and intellectual property. Therefore, the problems of cyberattacks and slow internet speeds are of the utmost importance to businesses and organizations, and effective solutions must be found to address them [17]. Various solutions are available to address the issue of cyberattacks and slow internet speeds, including upgrading hardware and software, optimizing network configurations, and deploying SDN and VNFs [18]. Additionally, recent research has explored the use of machine learning algorithms, edge computing, and implementing quality of service (QoS) and hybrid cloud architectures to optimize network performance and enhance incident response capabilities and cyberattack depletion [19]. Cyberattacks and sluggish internet speeds can have a substantial influence on incident response times, resulting in serious implications for businesses and organizations. Therefore, finding effective solutions to these problems is crucial. The integration of machine learning algorithms, edge computing, QoS implementation, blockchain technology and hybrid cloud architectures with SDN and VNF technologies can offer powerful and flexible solutions for improving network performance and incident response capabilities.

## 2. Related Work

This section discusses the salient features of existing approaches. Abubakar and Pranggono [20] proposed machine learning as a solution for SDN-based intrusion detection and prevention. They further explored and highlighted the benefits and challenges of the proposed approach. They concluded that although machine learning can improve the accuracy of intrusion detection and reduce false positives, challenges related to scalability and training data availability still exist. Ahmed et al. [21] proposed VNF chaining and network slicing as possible solutions. The authors also mentioned their respective benefits and limitations. Research on the VNF sphere was introduced by Wang and Zhao [22], who explored the use of edge computing to improve network performance and address the challenges of latency and bandwidth requirements in incident responses. The authors provided an overview of edge computing architectures, applications, and challenges, highlighting their potential to improve incident response times and reduce network congestion. Karakus and Durresi [23] contributed to the development of QoS in SDN networks and identified its potential to improve network performance and response times. They explored various QoS techniques and their effectiveness in addressing network congestion and improving the QoS in SDN. Another relevant study introduced by Li et al. [24] focused on blockchain-based collaborative software-defined networking (BCSDN) The authors proposed the use of blockchain technology in SDN to improve network security and reduce the risk of cyberattacks. They discussed the potential benefits of blockchain in providing a tamper-proof record of network activities and enhancing incident response capabilities. Yang et al. [25] provided an overview of current developments in network function virtualization (NFV) resource allocation. The authors generalized and examined four typical resource allocation issues: the VNF placement issue, the VNF placement and traffic routing problem, the VNF redeployment and consolidation issue, and the NFV traffic routing issue. Following that, two crucial quality of service (QoS) parameters—delay calculation models and VNF protection (availability) models—are investigated in NFV resource allocation.

Xu et al. [26] proposed a hybrid-assisted dynamic intrusion detection system (HADIDS) for improving network performance. This paper focused on the potential benefits of hybrid cloud computing in terms of scalability and cost efficiency and discussed the challenges related to security, privacy, and interoperability. Research in the sphere of VNF by Basu et al. [27] addressed the problem of limited network capacity and storage that can hinder QoS in a network. To optimize the placement of VNF instances over the service function chains (SFCs) for superior service delivery, the authors proposed a dynamic VNF sharing approach called FlexShare-VNF. According to Kim and Kim’s [28] research, the VNF placement approach was based on VNF characteristics and used information about each node’s resources to assign VNFs more efficiently. Furthermore, the authors suggested a method for identifying an appropriate node for placement through periodic searching of information concerning resource updates prior to VNF placement, subsequently assigning VNFs quickly upon request. Taniguchi and Shinomiya [29] proposed virtualized networks to minimize computing and network resources in the event of VNF failures. The proposed method aims to ensure sustainability against multiple VNF failures, which can cause significant damage to the network, by minimizing the cost of computing and network resources. The integration of VNFs with SDN technology can significantly improve the performance and efficiency of 6G networks. By leveraging the flexibility and programmability of SDN, VNFs can be dynamically deployed and managed to meet the specific requirements of different network functions and services. This approach improves resource allocation, reduces network congestion, and enhances security by enabling the implementation of advanced network policies and protocols. Yao et al. [30] proposed an anomaly detection with intrusion network framework (DINF). An anomaly detection approach leveraged both signature-based and anomaly-based techniques to enhance IoT devices. The authors recognized the limitations of using only one approach and suggested that combining them would lead to a more effective and efficient IDS. Their proposed system incorporated a signature-based approach to detect known attacks and an anomaly-based approach to identify unknown attacks. Zheng et al. [31] proposed a solution to mitigate the security risks associated with the Internet of Things (IoT) by dynamically creating and deploying firewalls based on the network traffic patterns. The solution employs machine learning algorithms to analyze network traffic patterns and identify potential security threats. The identified threats are then mitigated by dynamically creating and deploying firewalls on the affected devices in the IoT network. Table 1 shows comparison of the state-of-the-art approaches.

## 3. Proposed Efficient IoT Cyberattack Depletion Using VNFSDN and 6G

**Figure 2:**
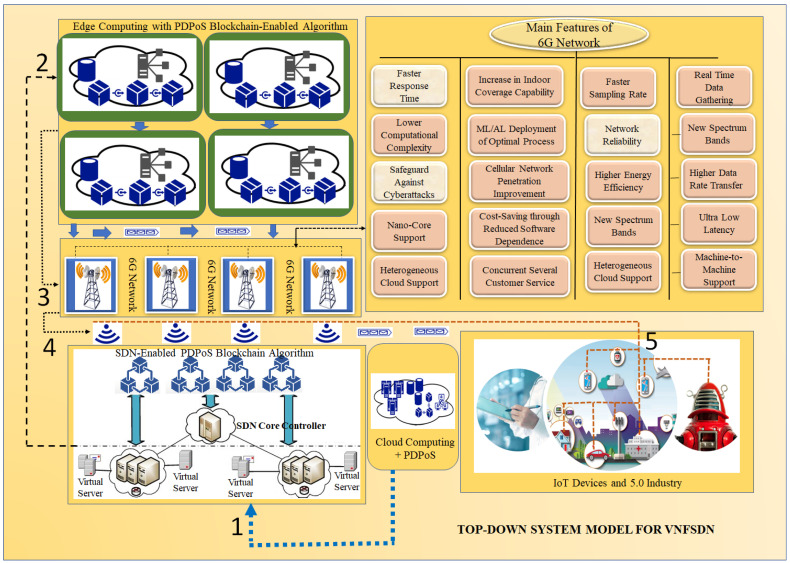
System model for cyberattack depletion using VNFSDN.

The proposed approach inherits the features of SDN, 6G technology, VNF, edge computing and the PDPoS consensus algorithm to enhance network security, as depicted in Figure 2 showing a top-down system model for IoT-enabled devices.

Each IoT network segment in our proposed solution will have several Instantiation Levels (ILs) as listed in [32]. To achieve the optimum resource allocation, the scalability will initially move between many ILs on just a single network segment. However, the application will be scaled out by deploying new instances of network segments rather than raising the overall number of VNF instances on a single segment when the network layer hits its maximum capacity and becomes congested. The concept and execution of scalability at the network segment level rather than the VNF level is our special contribution. The allocation of resources of each IoT segment will still be optimized while switching between several ILs. A single network segment’s design ultimately exceeds the upper limit of allowable traffic, which results in the failure of IoT services; hence, such a network design is crucial for service providers. The high service availability that a service provider urgently needs can be provided by the proposed architectural scheme. The proposed architecture makes it possible to dynamically increase the number of network segments in accordance with the volume of incoming data, freeing IoT services from the capacity restrictions of simply one network segment. On the other hand, 6G alone can face security concerns in intelligence network management implementation. The closed-loop network automation may present security vulnerabilities such as DoS, Man-In-The-Middle (MITM), and deception attacks for the IoT devices. DoS attacks can be carried out by progressively increasing the amount of fake high load in VNFs to improve the efficiency of virtual machines (VMs). MITM attacks can be carried out by simulating fault occurrences and intercepting domain control messages in order to reroute traffic through hostile IoT devices. Tampering with sent data can be used to carry out deception attacks. Second, if 6G networks employ intent-based interfaces similar to zero-touch network and service management (ZSM), which are susceptible to information leakage, unwanted configuration and aberrant behavior assaults may emerge for IoT devices. To address these issues, SDN allows network administrators to centrally manage the network and deploy virtualized security functions for IoT devices. SDN provides a flexible and powerful platform for developing VNFs for analyzing network traffic and detecting security threats for IoT devices. By leveraging the SDN and 6G, the proposed approach provides a scalable and efficient method for managing the security of IoT devices. Furthermore, blockchain technology is used; nevertheless, blockchain technology suffers because of its limitations in dealing with scalability. As a result, the PDPoS consensus algorithm is used to address the blockchain’s scalability and flexibility issues, resulting in proper security management of IoT devices. The aforementioned VNF deployment has several advantages.

**Figure 3:**
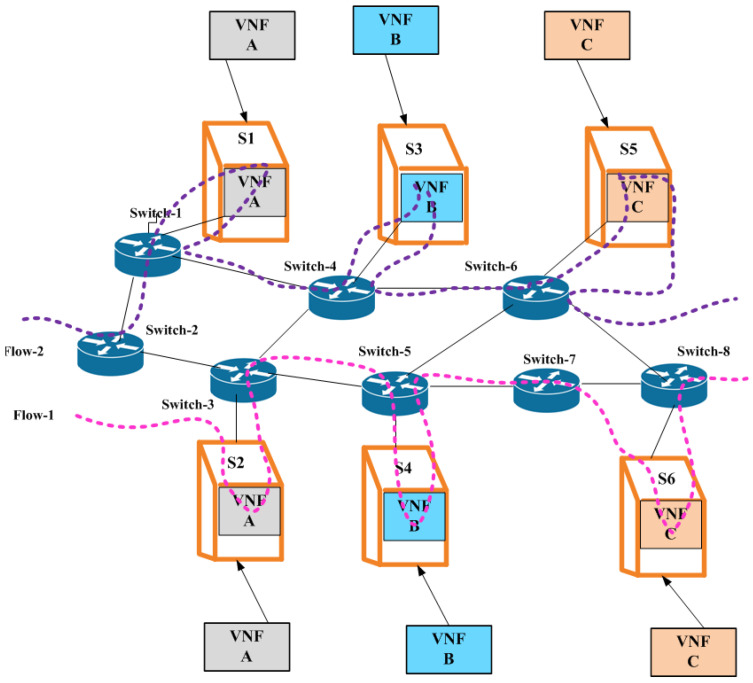
Traffic forwarding process, integrating SDN with VNF.

VNF does not require a unique piece of hardware for each service. The network services can be virtualized and quickly deployed in suitable places. The VNF eliminates the need for hardware and lowers hardware costs and energy usage in SDN. The technologies of SDN and VNF are separate but complimentary. Although they are not mutually exclusive, they work best when combined to their fullest extent, while VNFs virtualizes network tasks and deploys them as software on multipurpose high-capacity servers. The SDN centralizes the decision-making process in the network by utilizing the programming capabilities of forwarding devices. Both SDN and VNFs offer a flexible environment where traffic forwarding rules are influenced by the VNF location and operational requirements. In particular, the ability of SDN to swiftly and dynamically install particular routes to support service function chaining is a key characteristic used for VNF deployment. SDN would make it considerably simpler to deploy service functionality chains and route traffic flows across them. The VNF placement challenge is described as the task of determining the best locations for VNFs and the best distribution of traffic to those VNFs in order to satisfy their forwarding needs while making effective and efficient use of resources. However, as shown in Figure 3, the integration of VNFs with SDN maximizes computational power and network performance. Due to the VNF placement issue, which works in every case, energy consumption rises when more servers are running. The structure and capacity of the network, the power consumption of the servers, the amount of money available for software and energy use, the expectations for the user experience, and many other aspects all have a direct impact on how suitable a solution is. We only consider one trade-off between attaining load balancing objectives and lowering energy use: reducing the servers’ and the switches’ overall power usage. We employed a novel metric known as the Relation Degree (RD), which was suggested in [33]. This measure describes the service chain dependencies and interaction traffic. After determining the RD for each service chain pair, we deployed the function pairs on the same server or servers nearest to one another. It is a coordinated strategy that handles flows in a static manner while taking into account traffic data at the placement stage. The findings demonstrate that we may efficiently cut energy usage and average delay in transmission without sacrificing network performance.

This allows network administrators to deploy and manage security functions as virtualized services. In addition, costs are reduced, and the efficiency of network security is increased for IoT devices. The use of 6G for VNFs provides a flexible and customizable solution for analyzing network traffic and detecting potential threats for IoT devices. The centralized management provided by SDN allows network administrators to manage network resources through a single point of control. This leads to reduced complexity and improved overall network security for IoT devices. Overall, the proposed approach provides a powerful, efficient and secure solution for IoT devices that is well suited to the demands of modern businesses and organizations. The proposed solution consists of three main phases:

- Threat filtration process for IoT devices;
- Threat-capturing and decision-driven process for IoT devices;
- Modeling of a blockchain-enabled consensus algorithm for cyberattack depletion.

### 3.1. Threat Filtration Process for IoT Devices Using VNFSDN and 6G Technology

**Figure 4:**
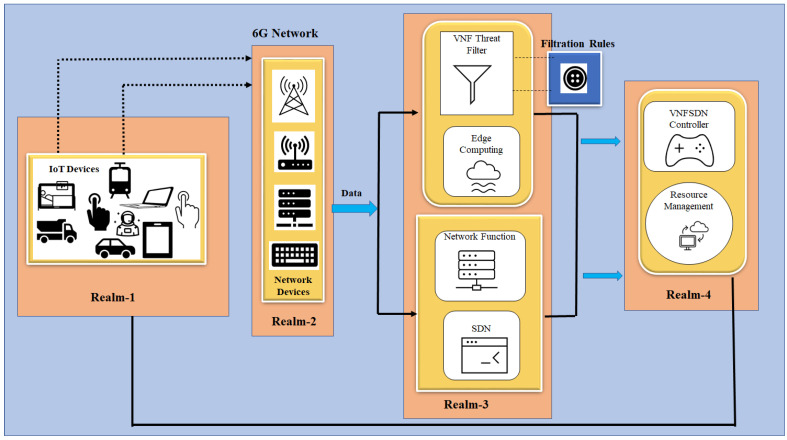
Threat filtration process using VNFSDN and 6G.

Here, we shows the process of securing 6G networks using VNFs and SDN technology. With the usage of 6G, networks can be autonomous and capable of self-configuration, self-monitoring, self-healing, and self-optimization (Self-X). The ongoing specification efforts incorporate artificial intelligence (AI)/machine learning (ML) as an inherent element in future networks including the ETSI ZSM architecture, requiring closed-loop operation, artificial intelligence and machine learning with ubiquitous automation of network management activities including security. However, cyber threats are unavoidable. Thus, there is a need of threat filtration to combat against emerging cybersecurity threats for IoT devices, as depicted in Figure 4. Algorithm 1 provides a threat filtration process which takes the VNF, SDN, network framework, protocol, packets, and router as inputs. It processes the data by transmitting them through SDN, network framework, VNF, and a router. The algorithm then checks whether the protocol used is secure; if not, the packets are blocked for IoT devices. If the protocol is secure, the packets are forwarded to IoT devices. Algorithm 1 continues looping when an SDN-to-VNF connection is active. Finally, the filtered traffic can be obtained, indicating that all packets are secure. The filtering process is described in detail in Algorithm 1.
**Algorithm 1:** Threat filtration process using VNFSDN and 6G technology**Input:** $\left\{P_{r},P_{c},V,S,F,R\right\}$ in**Output: $\left\{V\right\}$** out **Initialization:** *{V: Virtual network function; S: Software defined networking; $S_{c}$:* *Security; T: Transmitter; R: Receiver; F Framework; $P_{r}$: Protocol; $P_{c}$: Packet}* **Set** V, S, F, $P_{r}$, $P_{c}$, R **Do process:** S∈F→V∈R **Check** SandV are active **Determine** $P_{c}\in T$ **if** $P_{c}\neq S_{c}$ **then**   V→S Block $P_{c}$ **end if** **if** $P_{c}=S_{c}$ **then**   V→S forward $P_{c}$ **end if**

Algorithm 1 illustrates 6G security using VNF and SDN technologies. Step 1 gives the initialization of the variables. Steps 2–3 provide the input and output, respectively. Step 4 sets the values of the variables. Step 5 checks if S is a part of F. If so, V is added to R. Step 6 continues to the next steps as long as both S and V are active. Step 7 checks if whether the packet (Pc) is part of the transmitted packet (T). Steps 8–10 represent if the packet (Pc) is not equal to the security packet (Sc), and then the VNF (V) blocks the packet (Pc). Steps 11–12 represent the security process of the packet destined for IoT devices. If the packet is secure, then the packet is forwarded to the IoT devices. Steps 13–15 end with statements and algorithms, respectively. Figure 3 shows how the VNF and VNFSDN work together to ensure efficient traffic filtering and management. The VNFSDN controller receives the filtered traffic from the VNF and uses its intelligence to make decisions on how to route traffic through the network. Using the OpenFlow protocol, the VNFSDN controller communicates with the SDN switches and routers to ensure that traffic is efficiently forwarded to its destination. The controller can also adjust routing paths based on network traffic and congestion to ensure efficient traffic management for IoT devices. The threat filtration in Algorithm 1 has a time complexity of O (log n), which makes the threat detection operation much faster and helps in securing communication for IoT devices.

**Definition 1.**  *The 6G security with VNF and SDN technology is an algorithmic process that involves virtual network functions (V), software-defined networking (S), network frameworks (F), protocols ($P_{r}$), packets ($P_{c}$), routers (R), and security ($S_{c}$). This algorithm aims to ensure the security of transmitted packets in a 6G network environment. Calculation of secure traffic (in percentage) is shown in Equation (1).*

(1) $$S=\left(\frac{\sum P_{c}-\sum blP_{c}}{\sum P_{c}}\right)\times P_{r}$$

*where ($\sum P_{c}$) is the sum of the packets received, and ($\sum blP_{c}$) is the number of blocked packets. This equation can be used to determine the traffic security. $P_{r}$ denotes the percentage. This equation calculates the percentage of secure traffic ($P_{c}$) by subtracting the sum of the blocked traffic ($blP_{c}$) from the sum of all traffic ($P_{c}$), dividing the result by the sum of all traffic ($P_{c}$) and multiplying it by 100%. This equation is used to measure the effectiveness of the security measures in blocking unauthorized traffic. The effectiveness of the security mechanism can be evaluated using Equation (2).*

(2) $$T_{d}r=\left(\frac{\sum T_{d}th}{\sum T_{g}th}\right)\times P_{r}$$

*where ($T_{d}r$) is the threat detection rate, ($\sum T_{d}th$) represents the total number of detected threats, and ($\sum T_{g}th$) represents the total number of generated threats. A higher value indicates a more effective security mechanism. Equation (2) represents a method to measure the effectiveness of a security mechanism by calculating the threat detection rate ($T_{d}r$). The ($T_{d}r$) is determined by dividing the sum of the total number of detected threats by the sum of all generated threats.*

**Theorem 1.**  *The 6G security with VNF and SDN ensures the security of transmitted packets by blocking unauthorized access and only allowing authorized access.*

**Proof.**  The algorithmic process begins by initializing the necessary components of the 6G network environment. The input parameters are set to include the VNF, SDN, network framework, protocol, packet, and router. The output parameter is a packet (Pot) transmitted over the network. The filtering packets ($\sum F_{pk}$) are based on their security obtained from Equation (3).

(3) $$F_{pk}=\left\{\begin{array}{c} \int_{t=0}^{t=\infty }S\left(t\right)\to F\times O_{pc},\mathrm{if}O_{pc}\neq S_{l}\\ \sqrt{V\to S\times O_{pc}},ifO_{pc}=S_{l} \end{array}\right.$$

where ($S_{l}$) is the desired security level, F is the framework, and ($O_{pc}=S_{l}$) is represented by the integral of the limit of the software-defined networking (S(t)) as (*t*) approaches infinity and transitions to the network framework (*F*). This integral represents the process of blocking packets without the required security value. Algorithm 1 also prohibits unauthorized users from using the system. The accuracy of the algorithm can be calculated using Equation (4).

(4) $$U_{br}\left(A\right)=\left(\frac{\sum B_{uu}}{\sum U_{ua}}\right)$$

where $U_{br}\left(A\right)$ represents the user blocking rate, ($B_{uu}$) represent the sum of all blocked unauthorized users, and ($U_{ua}$) represent the total number of unauthorized user attempts. A higher ($U_{br}\left(A\right)$) indicates a more effective security mechanism. Equation (4) is used to calculate the accuracy of the algorithm by dividing the total number of blocked unauthorized users by the total number of unauthorized user attempts. This provides a measure of the effectiveness of the algorithm in preventing unauthorized access.    □

**Hypothesis 1.**  *Adding more routers will improve the secureness of traffic.*

**Proof.**  The secureness of traffic ($S_{t}$) can be calculated using Equation (5).

(5) $$F_{pk}=\int_{t=0}^{t=T}a_{n}\sqrt{S_{n}\left(t\right)}dt>\int_{t=0}^{t=T}a_{n}\sqrt{S_{1}\left(t\right)}dt$$

where (*S*) is the number of routers, (*T*) is the time duration, ($a_{n}$) is the impact of adding additional routers to the network, and ($S_{n}\left(t\right)$) is the security of the traffic in the network with n routers at time t. Assuming that the security is proportional to the square root of the number of routers in the network, it can be written as $S_{n}\left(t\right)=\sqrt{n}$.

By adding more routers, the first integral increases due to the positive impact ($a_{n}$). The integral of $\sqrt{S_{n}}\left(t\right)$ over time interval [0, T] represents the overall traffic security of a network with a single router. However, this security level is assumed to be lower than that of networks with multiple routers. As the number of routers in a network increases, the total traffic security also increases. By utilizing mathematical tools, such as limits and integrals, we can make the hypothesis more precise and easier to comprehend. Thus, the function $S_{n}\left(t\right)$ is calculated using Equation (6).

(6) $$S_{n}\left(t\right)=\left(\sqrt{S_{b}}+N_{I}Sin\theta \cdot Sin\left(W_{nt}\right)\right)$$

where $\sqrt{S_{b}}$ represents the baseline security of the network, ($N_{I}$) represents the impact of noise or other factors, and $sin(W_{nt}$) represents a sinusoidal oscillation with frequency ($W_{n}$) that can affect the security of the network. The equation presented here captures the dynamic nature of security, which can fluctuate over time owing to various factors, including the number of routers in the network and the presence of noise or variability in traffic. By incorporating these variables, the equation provides a more comprehensive and accurate assessment of the overall network security.

(7) $$Sin\left(W_{nt}\right)=\left(Sin\theta \cdot sin(m\sqrt{n_{t}})\right)$$

where (*m*) is a constant that represents the frequency of the sinusoidal oscillation. As the number of cycles n increases, the oscillation frequency increases proportionally, which can have implications on network security. As the number of routers increases, the oscillation frequency increases proportionally, which can affect network security.

Adding more routers can segment the network into smaller and more manageable subnets, which can help contain potential security breaches and limit the damage caused by attacks. The effectiveness of network segmentation ($E_{ns}$) can be evaluated using Equation (8).

(8) $$E_{ns}=\left(\frac{NT-Nsusp(pc)}{NT}\right)$$

where ($E_{ns}$) is the effectiveness rate of the network segmentation, NT is the total number of devices in the network, and Nsusp(pc) is the number of devices affected by the suspicious packets (Pc). A higher ER indicates a more effective segmentation strategy. To enhance access control policies and prevent unauthorized access to sensitive resources, it is possible to utilize additional routers. The efficacy of these measures is assessed using the following equation:

(9) $$AC_{ef}=\left(\frac{A_{t}-A_{f}}{A_{t}}\right)\times P_{r}$$

where ($AC_{ef}$) is the access control effectiveness rate, ($A_{t}$) is the total number of attempts to access a resource, and ($A_{f}$) is the number of failed attempts. A higher ($AC_{ef}$) value indicates a more effective access control mechanism. Incorporating additional routers into a network architecture offers essential redundancies and fail over capabilities. This, in turn, can help guarantee continuous network operation, even in the face of unexpected failures or malicious attacks. To assess the efficacy of the redundancy, Equation (10) can be used.

(10) $$R_{r}=\left(\frac{T-D_{t}}{T}\right)\times P_{r}$$

where ($R_{r}$) is the redundancy rate, (*T*) is the total time during which the network is operational, and ($D_{t}$) is the total downtime. A higher ($R_{r}$) indicates a more reliable and resilient network.    □

**Lemma 1.**  *Routers are capable of performing firewall tasks.*

**Corollary 1.**  *An increased number of routers results in a higher volume of monitored traffic, thereby increasing blocked packets ($B_{pc}$). The lack of suspicious packets $\varphi S_{pc}$ is a sign of greater security, as determined by Equation (11).*

(11) $$\varphi S_{pc}=\int_{0}^{T}\sqrt{M_{n}\left(t\right)}dt\to \infty ,as\;\;n\to \infty$$

*where ($M_{n}$) denotes the amount of traffic monitored at time (t) in a network of n routers. Equation (11) states that if the integral of the square root of the amount of monitored traffic ($M_{n}$) from zero to (T) (a specific time period) approaches infinity as n (the number of routers in the network) approaches infinity, then there is a greater level of security in the network if fewer suspicious packets are transmitted. In other words, if the network can handle a large amount of traffic without detecting any suspicious activity, it is considered more secure.*

(12) $$M_{n}\left(t\right)=\sum_{i=0}^{n}f\left(Pc_{i}\right)\left(t\right)*\left(Pc_{i}\right)\left(t\right)$$

*where $\left(Pc_{i}\right)\left(t\right)$ represents the (i) packet in the network at time (t). The sum of (i) represents the total amount of traffic being monitored by (n) routers in the network. This equation represents a mathematical model for monitoring the network traffic, where ($M_{n}\left(t\right)$ is the total traffic monitored by (n) routers in a network at time (t). The sum of (i) represents the contribution of each packet to the monitored traffic, where $\left(pc_{i}\right)$ represents the (i-th) packet in the network at time (t), and $f\left(Pc_{i}\right)\left(t\right)$ is a function that maps the properties of the packet to a weight that reflects its importance in the monitored traffic.*

### 3.2. Threat-Capturing and Decision-Driven Process for IoT Devices

Threat capturing is the process of intercepting and recording network traffic for the IoT devices. This is useful for analyzing the network behavior, troubleshooting issues, and identifying threats to IoT devices. Threat capturing in VNFSDN involves capturing network threats at a specific point in the network, extracting the necessary data, and processing the data for further analysis. Threat capturing in VNFSDN offers several advantages over traditional threat-capturing methods. First, it allows the capture and processing of threats at specific points in the network, which can provide greater visibility of the network behavior. Second, it allows the capture of threats according to specific criteria, which can help filter out noise and focus on specific areas of interest. Finally, it allows packets to be captured at the VNF level, which can provide more granular insights into network behavior and performance. Algorithm 2 represents the process of threat-capturing and saving packets for further analysis. First, the variables and parameters required for capturing and storing threat packets are initialized. The user interface, MAC address of the access point, and other inputs are accepted. The output of the algorithm is a file that is produced and recorded. The Linux network-monitoring tool is deployed and configures the network resources. To create the final threat-captured file, the method gathers and adds packets to previously collected packets. For subsequent investigations, the recorded packets are saved to a designated folder. The whole loop ensures ongoing packet capture and storage until the process is complete.
**Algorithm 2:** Threat-capturing and -mitigating processes using VNFSDN**Input:** $\left\{P_{r},P_{c},V,S,F,R\right\}$ in**Output: $\left\{V\right\}$** out **Initialization:** *{*$N_{c}$: Network Chanel; Mpa: MAC address of access point; *I*: Interface; Pcf: Packet-captured file; $L_{t}$: Linux tool; $N_{m}$: Network monitoring; *N*: Network; $F_{d}$: Folder; $P_{c}$: Packets} **Set** $N_{c},Mpa,I$ **Do process** $N_{m}\in N\leftarrow Lt$ **while** $N_{m}\in N\leq 0$ **do**  **Capture** P  **Sum** P+1  **Do** $N_{m}=0$  **Save** $Pcf=F_{d}$ **end while**

**Figure 5:**
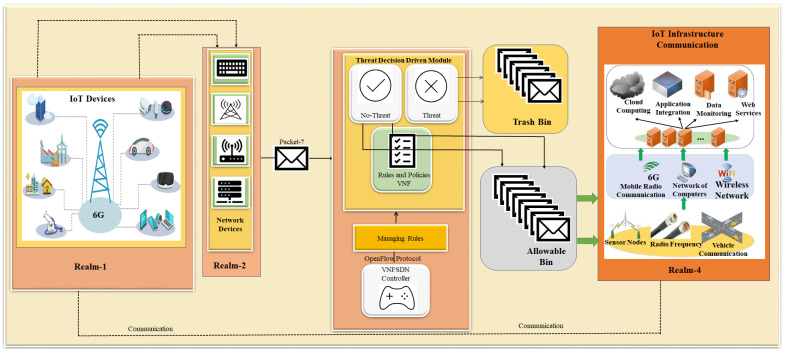
Threat-capturing and decision-driven processes using VNFSDN.

The network traffic for IoT devices is analyzed by the VNFs to make decisions about the threats to IoT devices. The SDN controller then receives these alerts and makes decisions based on predefined response policies. These policies can be mathematically represented as a set of conditional statements that consider factors such as the severity of the threat, the location of the affected network resources, and the overall state of the network. Figure 5 depicts the VNFSDN and 6G technology-based threat collection and decision-making process. The process begins with a network packet being transmitted through the network. The SDN component is then used to capture packets and extract the necessary data. Threat-capturing and -mitigating processes are of paramount significance, as illustrated in Algorithm 2.

Algorithm 2 illustrates packet capture and saving using the VNFSDN approach. Step 1 gives the initialization of the variables, including the network channel (Nc), MAC address of the access point (Mpa), interface (*I*) used for capturing packets, packet-captured file (Pcf), Linux tool (Lt) used for network monitoring, network monitoring ($N_{m}$), the network (*N*) being monitored, the folder ($F_{d}$) where the captured packets will be saved, and the packet (P) being captured. Steps 2–3 represent the input and outputs, respectively. Step 4 sets the variables. Steps 5–11 represent the packet-capturing and -saving processes. The algorithm enters a loop that captures packets, adds each captured packet to a packet-captured file, and resets the network monitoring ($N_{m}$) to zero. This loop continues until network monitoring ($N_{m}$) ceases within the network (*N*) being monitored. When the loop ends, the packet-captured file (Pcf) is saved in a designated folder ($F_{d}$). The time complexity of the threat capture algorithm is O (log n), which makes the threat detection procedure substantially faster.

**Definition 2.**  *A VNF is a software component of SDN that performs specific network functions, such as firewalls and load balancers, on a network framework F. The VNFs communicate with standardized protocols ($P_{r}$) using packets $P_{c}$ routed through the network R. VNFs enable dynamic and scalable network architectures to adapt to changing business requirements and traffic patterns.*

**Theorem 2.**  *The proposed algorithm for the packet-capturing and -saving processes can efficiently capture and store network packets.*

**Proof.**  The algorithm begins by initializing the necessary variables, such as the network channel, MAC address of the access point, interface, packet-captured file, Linux tool, network monitoring, network, folder, and packets. It then begins the network-monitoring process using the Linux tool. When the network-monitoring variable is less than or equal to one, the algorithm captures packets from the network channel and adds them to the packet-captured file. The captured packets are then summed to calculate the total number of captured packets. After capturing the desired number of packets, the network-monitoring variable is set to zero to stop the network-monitoring process. The packet-captured files are saved to a designated folder. The total number of packets captured is given by

(13) $$|P_{p}|=\sum_{i=1}^{N_{d}}\gamma \pi \sum_{j=1}^{N_{d}}||\delta -\delta^{-}\left|\right|$$

where $\delta -\delta^{-}$ represents the time when generating the packets, Nd is the desired number of network packets to capture, $P_{p}$ is the set of captured packets, and (γπ) is an indicator variable that takes the value of 1 if the packet is captured and 0 otherwise. The time required to capture $N_{c}$ packets is given by

(14) $$t_{c}=\left(\frac{TP_{c}\left(a\right)}{N_{c}+P_{c}\left(t\right)}\right)$$

where *t* is the time required to capture the total number of available packets, and $TP_{c}\left(a\right)$ is the capacity of the network channel. Equation (14) calculates the time required to capture a total number of packets $t_{c}$ in a network channel with a given capacity $N_{c}\left(t\right)$ and the time taken to process each packet $P_{c}\left(t\right)$. This formula suggests that the time required to capture packets increases with the number of packets and the time required to process each packet. This also shows that a network with a higher capacity can capture packets faster than a network with a lower capacity.

The efficiency *E* is defined as the ratio of the number of packets captured $\left(|P_{p}|\right)$ to the time required to capture them *t*.

(15) $$E=\left(\frac{\left(|P_{p}|\right)}{t}+\frac{C_{v}*\left(|Pp|\right)}{TP_{c}\left(a\right)}\right)$$

Equation (15) suggests that efficiency can be improved by increasing the number of packets captured, reducing the time required to capture them, or adjusting the constant $C_{v}$ or the total number of available packets $TP_{c}\left(a\right)$. The network-monitoring variable is less than or equal to 1 while the algorithm captures packets from the network channel. Therefore, the maximum number of packets $Max_{P_{c}}$ that can be captured in a single iteration is given by

(16) $$Max_{P_{c}}=\left[min\left(C_{v}\right),TP_{c}\left(a\right)-\left(\right|P_{p}\left|\right)\right]$$

where $Max_{P_{c}}$ represents the maximum number of packets captured without exceeding the desired total number of packets. The captured packets are then added to packet-captured files. Therefore, the file size $F_{s}$ is in bytes after capturing the packets.

(17) $$F_{s}=\left(F_{in}+\left(\right|P_{p}\left|\right)*\lambda P_{c}\right)$$

where $F_{in}$ is the initial file size and ($\lambda P_{c}$) is the average size of a network packet. This equation calculates the file size in bytes after capturing n packets and adding them to the initial file size $F_{in}$. The average size of a network packet is denoted as ($\lambda P_{c}$), which is multiplied by the number of captured packets $P_{p}$ and added to the initial file size. The algorithm efficiently captures and stores network packets by iteratively capturing the maximum number of packets possible while the network-monitoring variable is less than or equal to 1 and adding them to the packet-captured file until the desired number of packets is captured. □

**Hypothesis 2.**  *For effective network analysis and incident responses, a real-time packet capture and saving algorithm is anticipated to deliver precise and timely network packet data.*

**Proof.**  The algorithm continually captures and stores network packets in real time using a network-monitoring process. It is suitable for real-time network packet analysis owing to its capability to enable instant access to network packets for analysis and troubleshooting. Packet-capturing and -saving processes are given by

(18) $$\Delta P_{c}=\left(\Delta \left(P_{re}\right)P_{c}+1\right)$$

where $\Delta P_{c}$ is the count of the captured packets, $\Delta \left(P_{re}\right)P_{c}$ is the previous count, and 1 is added to the previous count for each captured packet. Equation (18) represents the process of packet capturing and saving, in which the count of captured packets $\Delta P_{c}$ is calculated by adding one to the previous count $\Delta \left(P_{re}\right)P_{c}$ of each captured packet. It is a simple equation used to track the number of packets captured by a network-monitoring or security tool. This equation provides a straightforward method for counting and tracking the number of packets captured during packet capturing. The packet loss rate $P_{lr}$ is given by

(19) $$P_{lr}=\left[\frac{\sum P_{c}\left(\psi dr\right)}{\sum P_{c}}\times P_{r}\right]$$

where $\sum P_{c}$ is the sum of the packets, and (ψdr) denotes the total number of dropped packets. In this case, it represents packets dropped owing to network congestion or other reasons. The $P_{lr}$ measures the percentage of packets that are not successfully delivered to a destination. The $P_{c}\varpi$ measures the percentage of packets that are not successfully delivered to their destination. This does not help network administrators identify areas of the network that may require optimization or additional resources to reduce packet loss. The network latency $N_{lt}$ is given by

(20) $$N_{lt}=\left[\frac{1}{\sum P_{c}}\times \sum_{i}\left(D_{t}-A_{t}-P_{t}\right)\right]$$

where ($A_{t}$) is the arrival time, ($D_{t}$) is the departure time, and ($P_{t}$) is the processing time. The processing time ($P_{t}$) is the time required to process packet i using the network. This metric is particularly useful for identifying any areas in the network that may be experiencing delays or bottlenecks and can be utilized to optimize both routing and network configuration. By measuring and analyzing the processing time, network administrators can gain a better understanding of how their network functions and make informed decisions to improve their overall performance. Thus, network throughput $\left(\forall N_{th}\right)$ with 6G can be obtained as follows:

(21) $$N_{th}=\left(\frac{\sum P_{c}}{t_{t}}\right)$$

where the total time taken $t_{t}$ is the time required for all the packets to be transmitted. This metric is useful for quantifying the amount of data that can be transferred over a network within a specific period, and can be used to identify network segments that require improvements in data transfer rates. By analyzing $t_{t}$, one can gain valuable insights into network performance and identify areas where optimization efforts may be necessary. The network utilization $\left(N_{u}\right)$ is given by

(22) $$N_{u}=\left(\frac{T_{bn}}{B_{a}}\right)\times P_{r}$$

where the total bytes $T_{bn}$ is the total number of bytes transmitted over the network, and the available bandwidth $B_{a}$ is the maximum bandwidth the network can support. This metric measures the percentage of available bandwidth being used and can help identify areas of the network where traffic congestion may occur. Thus, the jitter (J) is given by

(23) $$J=\left(\frac{1}{P_{c}\left(t\right)}\right)\times \sum_{i=1}^{P_{c}\left(t\right)}{\left(P_{c}T_{a}+AP_{c}T_{a}\right)}^{2}$$

where $P_{c}T_{a}$ is the packet arrival time, $AP_{c}T_{a}$ is the average packet arrival time, $P_{c}\left(t\right)$ is the total number of packets, and the average packet arrival time is the average time between packet arrivals. Jitter measures the variation in packet arrival times. This can help identify areas of the network where delays or interruptions may occur. The packet delivery ratio $\left(P_{dr}\right)$ is given by

(24) $$P_{dr}=\left(\frac{\sum P_{sd}}{\sum \;P_{ts}}\right)\times P_{r}$$

where $P_{sd}$ is the number of successfully delivered packets and $P_{ts}$ is the total number of packets sent. This metric measures the percentage of packets that are successfully delivered to their destination and can be used to evaluate the overall performance of the network. The average packet size $P_{as}$ is given by

(25) $$P_{as}=\left(\frac{T_{bn}}{P_{ct}}\right)\times P_{r}$$

where $T_{bn}$ is the number of packets transmitted over the network. This equation can be used to optimize network performance by adjusting packet size limits or optimizing the network configuration. □

**Lemma 2.**  *The proposed algorithm for packet capture and saving is suitable for a real-time network packet analysis.*

(26) $$P_{sd}=\left(\frac{P_{d}}{P_{ct}}\right)\times P_{r}$$

*where $P_{sd}$ measures the percentage of packets that successfully reach their destination and provides an indication of the reliability of the network, and $P_{ct}$ denotes total number of the packets. The packet size has a great impact on the performance of the network, particularly when using 6G. Thus, the packet size $P_{c}\left(s\right)$ can be determined as follows:*

(27) $$P_{c}\left(s\right)=\left(H_{s}+PY_{s}\right)$$

*where the header $H_{s}$ size includes the protocol header, address, and control information, and the payload $PY_{s}$ size includes the actual data being transmitted. Network administrators can optimize network performance and ensure that network resources are effectively used by analyzing the packet size.*

The network throughput can be greatly improved by employing features (Ω) of 6G. Additionally, the administrators can identify bottlenecks and optimize the network to improve its performance. Thus, the optimized throughput $Th_{ot}$ can be determined as follows:

(28) $$Th_{ot}=\left(\frac{D_{ta}\times \Omega }{t_{t}\left(l\right)}\right)$$

where the amount of data transferred $D_{ta}$ is measured in bits or bytes, and latency *l* is the time delay between sending and receiving packets. The network administrators can assess network performance and identify potential issues with network latency or packet loss. Thus, there is a need to calculate the round trip time $T_{tt}$, which can be determined as follows:

(29) $$R_{tt}=\left(t_{2}-t_{1}\right)$$

where $t_{2}$ is the time taken for a packet to travel from the sender to the receiver, and $t_{1}$ is the time taken for the packet to travel back again, which is also known as the round trip time or RTT. By measuring the RTT, the following can be obtained:

- For each historical sample, a vector derived from the efficiency of the CPU, the main memory, and the number of VMs is achieved. All three parts have the same significance level.
- The PM on the cloud calculates the Euclidean distance between the current state and the best settings. The configuration is said to be optimal or acceptable when it is within the range of efficiency accepted by the PM of the cloud server. These settings are stored to meet efficiency requirements.
- The PM also calculates the distance of the total configuration to the total shutdown of the PM state for the cloud.

Let us assume that user *U* has the minimal distance from user position $U_{p}$ to join the edge computing. Thus, the distance matrix $U\times U_{p}$ between the mobile user and $U_{p}$ can be formulated as

(30) $$D\left(U\times U_{p}\right)=\left\lceil \begin{array}{c} dU_{1},Z_{1},dU_{1},Z_{2},\ldots ,dU_{t},Z_{n}\\ dU_{2},Z_{2},dU_{2},Z_{2},\ldots ,dU_{t},y_{n}\\ .\\ .\\ dP_{1},z_{1},dP_{2},z_{2},\ldots ,dP_{t},z_{n} \end{array}\right\rceil$$

Additionally, the positions of two mobile cloud nodes are denoted by *a* and *b*, and the nearest distance between two mobile cloud nodes d(m,n) is measured using the Euclidean distance.

Additionally, a user’s coordinates are indicated by the *x* and *y* …values. There, the closest distance can be determined, d (ϵ,ξ)

(31) $$d\left(\epsilon ,\xi \right)=\sqrt{{\left(\epsilon_{x}{-\xi )}_{x}\right)}^{2}+{\left(\epsilon_{b}-\xi_{b}\right)}^{2}}$$

The threshold distance identifies the range of the user’s participation in edge computing $E_{c}$ because $P_{ctr}$ represents the packet transmission from the user to the edge computing. Additionally, the distance between *U* and the edge computing performance is denoted by $\left(E_{c}-\alpha \right)$, and the distance between $E_{c}$ and user *U* is represented by $\left(E_{c}-\alpha \right)$. The entire user performance with 6G is associated with $E_{ci}$ and $E_{cj}$, and the systems are indicated by $S_{a}$ and $S_{b}$. The distance-determining fitness functions can be calculated as follows:

(32) $$P_{ctr}=\left(\frac{P_{ctri}}{P_{ctrj}}\right)$$

(33) $$P_{ctri}=\sum_{t=1}^{C_{a}}\left[\left|\right|S_{ai}-\alpha ||+\sum_{j=1}^{C_{b}}\left|\right|S_{aj}-\varpi \left|\right|\right]$$

(34) $$P_{ctrj}=\sum_{t=1}^{C_{a}}\sum_{j=1}^{C_{b}}||\varpi -\varpi^{-}\left|\right|$$

where $\varpi -\varpi^{-}$ represents the distance between two users.

**Corollary 2.**  *The ability of Algorithm 2 to capture and store network packets in real time can be useful for quickly detecting and responding to security incidents by providing access to important network data for analysis and troubleshooting. The efficiency of the proposed algorithm in capturing and storing network packets, E(efficiency), is directly proportional to the ability to detect and respond to security incidents, $R_{e}$ (response).*

(35) $$E=\left(K\times R_{e}\right)$$

The proportionality constant *K* represents the scaling component of the relationship and can be used to express the proportional relationship between *E* and $R_{e}$. This implies that as the ability to detect and respond to security incidents increases, the efficiency of the algorithm in capturing and storing network packets also increases proportionally.

Let $V_{t}$ be a vector representing the set of network resources and a scalar representing the current time. Let S(x,t) be a function that takes the inputs and returns the security status of the network resources. Based on S(x,t), a decision function d(S(x,t)) can be defined as follows:

(36) $$d\left(§\left(x,t\right)\right)=\left\{\begin{array}{c} \;1,if\;\;threat\;is\;detected\\ 0,\;therwise \end{array}\right.$$

If the security status of the network resources indicates the presence of a threat, the decision function outputs a value of 1 to indicate that a threat has been detected. Otherwise, the decision function outputs a value of 0, indicating that no threat has been detected. This decision function can be used in various security applications, such as intrusion detection systems, to automate the processes of threat detection and response. By continuously monitoring the security status of network resources and applying a decision function, security systems can quickly and accurately detect threats and take appropriate actions to mitigate them. If the decision function returns a value of 1, the SDN controller mitigates the security threat. This can be mathematically represented as

(37) $$a\left(x,t\right)=\sum b_{i}\left(x,t\right)\times d\left(S(x,t)\right)$$

where $b_{i}\left(x,t\right)$ is a binary function that maps the set of network resources that are affected by the security threat. The function $b_{i}\left(x,t\right)$ is defined as

(38) $$b_{i}\left(x,t\right)=\left\{\begin{array}{c} \;1,if\;\;network\;resource\;is\;affected\\ 0,\;therwise \end{array}\right.$$

Once the affected network resources have been identified, the SDN controller can take action to mitigate the threat. This may involve rerouting traffic, isolating the affected resources, or deploying additional security measures. In addition to responding to security threats, the VNFSDN approach enables proactive monitoring of the network to detect potential vulnerabilities before they can be exploited. This can be achieved using machine learning algorithms to analyze network traffic and identify patterns of behavior that may indicate an attempted attack. Mathematically, this can be represented as

(39) $$p\left(x,t\right)=\int S\left(x,t\right)$$

where P(x,t) represents the probability of a security threat occurring given the current security status of the network resources represented by S(x,t).

Let us assume that there are two system placed on the edge computing. The consumers are competing for access to them. A fundamental model of competition is created and combines the fuzzy fractional aspects of an ordinary differential equation with requests from the users to access the first system placed on the edge computing $\frac{dr_{ui}\left(t\right)}{dt}$ and the second system placed on the edge computing $\frac{dr_{uj}\left(t\right)}{dt}$.

(40) $$\frac{dr_{ui}\left(t\right)}{dt}=c_{ri}r_{ui}\left(t\right)\left(1-\frac{r_{ui}\left(t\right)}{M_{q}1}-\eta \frac{r_{ui}\left(t\right))}{M_{q}2}\right)$$

(41) $$\frac{dr_{uj}\left(t\right)}{dt}=c_{rj}r_{uj}\left(t\right)\left(1-\frac{r_{uj}\left(t\right)}{M_{q}2}-\hat{\eta }\frac{r_{uj}\left(t\right)}{M_{q}1}\right)$$

where $M_{q}1$ is the maximum capacity of dealing with the requests of the first system of the edge computing using the 6G network; $M_{q}1$ is the maximum capacity of entertaining the requests of the second PM of the MCC; $c_{ri}$ is the number of requests to connect with the first system; $c_{rj}$ is the number of requests to connect with the second system; $r_{ui}\left(t\right)$ is the request of each user using 6G within a given time; η is the competing capability of the user for the first system; and $\hat{\eta }$ is the competing capability of the user for the second system. The capabilities of the users depend on the characteristics of SDN and 6G because each system only needs a short amount of time to respond to the requests indicated by the user’s capabilities. Because each request from the user is processed with a distinct time delay, each system’s properties vary slightly from one another.

Suppose that $\lambda_{1}$ and $\lambda_{2}$ represents the latency of each system placed on edge computing. $\epsilon_{p1}$ and $\epsilon_{p2}$ indicate the maximum capabilities of the first and second systems, respectively. Thus, the complete model to deal with requests for the systems can be determined as follows:

(42) $$\frac{dr_{ui}\left(t\right)}{dt}=c_{ri}r_{ui}\left(t\right)\left(1-\frac{r_{ui}\left(t-\lambda_{1}\right)}{M_{q}1}-\eta \frac{{\left(r_{ui}\left(t-\lambda_{2}\right)-\epsilon_{p2}\right)}^{2}}{M_{q}2}\right)$$

(43) $$\frac{dr_{uj}\left(t\right)}{dt}=c_{rj}r_{uj}\left(t\right)\left(1-\frac{r_{uj}\left(t-\lambda_{1}\right)}{M_{q}2}-\eta \frac{{\left(r_{uj}\left(t-\lambda_{2}\right)-\epsilon_{p1}\right)}^{2}}{M_{q}1}\right)$$

The probability of a security threat occurring at a specific time based on the current security status of the network resources should be measured. By monitoring the network and analyzing behavioral patterns, both algorithms can be used to update the security status of the network resources and improve the accuracy of the probability calculations. Overall, the third phase of the VNFSDN approach plays a critical role in ensuring the effectiveness and efficiency of the network security solution. By leveraging the power of SDN and VNFs, this approach enables quick and targeted responses to security threats while also providing proactive monitoring to detect potential vulnerabilities. Mathematical representations of the decision-making process and proactive monitoring illustrate the potential of the VNFSDN approach to enhance network security in a scalable and efficient manner.

### 3.3. Modeling of the Blockchain-Enabled Consensus Algorithm for Cyberattack Depletion

**Figure 6:**
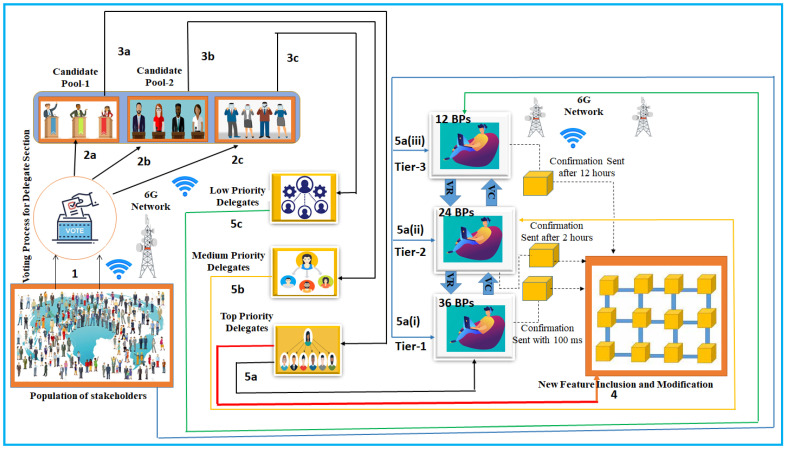
PDPoS consensus process for faster response and cyberattack depletion.

Prioritized delegated proof of stake (PDPoS) is used to counteract attacks to IoT devices. A predetermined number of sensor nodes or IoT devices which handle communication and oversee the ledger can be determined by PDPoS. As a result, these IoT devices create blocks and perform validation to stop any illegal transactions from being made by the attacker. The participants (IoT devices) are chosen through a voting process, and they have the option of being replaced or expelled if they act inappropriately or perform poorly. PDPoS asserts that it is more effective, scalable, secure and adaptable. The PDPoS is a modification to the current DPoS consensus that allows blockchain to have a three-tier network on top of the network, as depicted in Figure 6. PDPoS is a mechanism that is more flexible and scalable. Blockchain networks may be able to manage additional users, applications, and transactions without compromising decentralization or security. Moreover, for a PDPoS chain, the possibility for staked asset values to rise in proportion to the network value exists. In other words, the network’s economic security improves as the native token of the PDPoS chain increases in value. PDPoS has a scaling benefit over PoW because of this characteristic. Let $U_{b}$ be the number of blockchain users in the network, and when the number of the users increases there is the possibility of increasing the level of contention $\Gamma l_{c}$ that requires a level of consistency *l*. Thus, the scalability of the blockchain $S_{bc}$ can be balanced by employing PDPoS, which is given by

$$S_{bc}==\left(\frac{U_{b}}{1+\Gamma l_{c}\left(U_{b}-1\right)+C_{l}U_{b}\left(U_{b}-1\right)}\right)$$

In our proposed PDPoS, we define top-, medium-, and low-priority delegates. Our supposition is that an IoT device completes a communication on a platform powered by blockchain. Any of the tiers (tier 1, tier 2, and tier 3) may receive it for processing from the IoT device.

The response time for a Tier 1 transmission, whether it be to a user device or delegate, is only 100 ms at most. For the confirmation, Tier 2 can take up to two hours longer than Tier 3, which takes up to twelve hours. On the other hand, top-priority delegates can only communicate with Tier 1 block producers (BPs). The medium-priority delegates can communicate with Tier 2. The low-priority delegates can communicate with Tier 3 block producers. Tiers 1–3 have 36, 24, and 12 BPs, respectively. Tiers 2 and 3 are also responsible for the consensus, but the transaction cannot be confirmed, and the user device would be paying more to send the transmission straight to Tier 1. Every transmission on Tier 3 is sent to Tier 2 and would ultimately become part of Tier 1. This kind of transmission can be confirmed after 12 h. BPs from Tiers 3 and 2 cannot participate in Tier 1 at the same time, and vice versa. The incentive for validating would be reduced because Tiers 2 and 3 possess medium- and low-priority delegates. By clearing up Tier 1, the proposed PDPoS consensus mechanism would increase the network’s communication speed. Additionally, it enables consumers to pay less for blockchain benefits. The IoT-enabled user would be able to pay according to the urgency and use case under this arrangement. When a IoT-enabled device moves from Tier 3 to Tier 2 and then finally Tier 1, they can track their transmission and receive updates. The transmission will be sent directly to Tier 1 and obtain a timely confirmation within 100 ms, but the IoT-enabled device will have to pay an additional fee if they need the transaction completed right away. The approach will use a third-party database to store the hash in case a transaction from Tier 2 or 3 to Tier 1 is missing or fails. One needs a certain level of processing power and to keep a stake for a set amount of time in order to be a BP on Tier 1. After the allotted time has passed, the BPs would enter the election pool, where residents would vote on them every two hours. The top 6 block producers, out of a total of 36, would be known as Super BPs. A minimum of 30 BPs must validate a transaction for it to be considered final, and at least 5 of the 6 super BPs must sign and confirm the transaction in order for it to be considered final. This will increase the security and effectiveness of the network. Elections serve to determine the fate of the BPs for the following two hours every two hours. The same technique can be used for Tiers 2 and 3, with the exception that there will only be 20 and 10 BPs, respectively, and the Super BP concept will be dropped. An election would be performed every two hours.

Every PDPoS IoT-enabled device receives a reward. Both Super BPs and BPs are paid based on the number of blocks confirmed per hour. Voting rewards will also be given to the voters. This network could be utilized for high-frequency instantaneous transactions in a wide range of IoT-enabled applications (including commercial, health, and vehicle validation). PDPoS reduces the number of nodes necessary for the consensus and allows for faster and less expensive transactions, which aids in energy conservation for IoT devices. Energy consumption has been a major concern in traditional blockchain technology, but it has been addressed using this algorithm. PDPoS, on the other hand, has weaknesses due to its reliance on a small number of incorruptible delegates; however, in the presence of edge computing and a 6G network, it does not threaten the network’s decentralization and security. Due to this obstacle, small members with less impact in the network may find it difficult to enroll and engage in the network. However, the goal of this consensus algorithm is to prevent cyberattacks on IoT devices.

Another objective by employing this algorithm is to achieve stability.

Let $P_{t}$ be the participants and $Q_{t}$ be the transactions of the stable communication at time *t*. Given a finite time t={1,2,3,…,n}, the goal is to minimize the loss function $l_{\gamma }$, which can be determined as follows:

(44) $$l_{\gamma }=\sum_{i=1}^{n}{\left(d_{p}P_{t}\right)}^{2}+{\left(\Psi_{wd}Q_{t}\right)}^{2}$$

where $d_{p}$ denotes the decentralized process, and $Psi_{w}$ denotes the proportionate weight assigned to the trade-off between participants and price stability.

The PDPoS algorithm uses a balancing token to prevent unpredictability of the IoT devices. Each token displays consistency in its operation. Furthermore, there is a common pattern that allows for real-time monitoring in order to maintain the PDPoS predictable condition.

The quantity of tokens in circulation $T_{c}$ increases as tokens are awarded $T_{a}$ as rewards to nodes, while it decreases when tokens are reissued. Thus, the token-increasing process $T_{c}+1$ can be determined as follows:

(45) $$T_{c}+1=\left(T_{c}+T_{a}\right)-\left(\frac{C_{t}^{p}}{T_{t}^{cp}+A_{s}^{po}}\right)$$

where $C_{t}^{p}$ denotes the amount the controller’s account pays to active token holders to buy their tokens. This implies that the number of tokens purchased at time t is equal to H divided by the cost of the purchase.

$T_{t}^{cp}$ denotes the token’s current market price, and $A_{s}^{po}$ is the additional sum that the IoT-enabled user pays over the market price to entice them to sell their tokens. If the owners of the tokens are not rational, $A_{s}^{po}$ is equal to zero. Because a reasonable owner might not sell their tokens if they think the price would go up in subsequent timestamps, the IoT-enabled user could be required to offer an additional incentive price $A_{s}^{po}$ to disprove the agents’ predictions. To determine $A_{s}^{po}$ for a rational strategy, we developed a mathematical strategy.

Two stocks are kept by the IoT-enabled user, one of which is made up of tokens and the other of dollars. The values of these two stocks at time *t* are provided by $S_{t}^{tk}$ and $S_{t}^{U_{d}}$, respectively. The dollar balance rises as a result of the user purchasing tokens and falls as a result of market buybacks.

(46) $$S_{t}^{U_{d}}+1=S_{t}^{U_{d}}-\left(C_{t}^{p}+\delta t_{p}\right)$$

where the block is represented by the new variable $\delta t_{p}$. Hence, $\delta t_{p}$ is a linear function of the system’s total number of users. Users need to be validated before joining the blockchain. The block $S_{t}^{tk}$ is comparable to the security parameters that are stored within in, meaning it grows with the number of the newly joined users and shrinks with a decrease in users.

(47) $$S_{t}^{tk}+1=\left(S_{t}^{tk}-T_{a}\right)+\left(\frac{C_{t}^{p}}{T_{t}^{cp}+A_{s}^{po}}\right)$$

Thus, it is important to determine blocks in transit to avoid any kind of potential threat.

(48) $$A_{s}^{po}=\left(\frac{D_{t}^{t}}{T_{c}}\right)$$

where $D_{t}^{t}$ denotes the release of blocks at a given time.

The relationship between the number of IoT-enabled users in the system $S_{c}$, the block’s value $A_{s}^{po}$, and the demand for the block is (unspecified). Assuming that the release of the block is proportionate to the number of IoT-enabled users in the existing network at a specified time, it is possible to state that for a certain block value $\delta t_{p}$ ∝ $D_{t}^{t}$. The PDPoS algorithm completely models the value of each block in each phase. The transmitting block remains invariant when confirmed using Equations (46)–(48).

(49) $$\delta t_{p}=C_{t}^{p},\;\;\;\;\;\;\;\;\;\;\;T_{a}=\left(\frac{C_{t}^{p}}{T_{t}^{cp}+A_{s}^{po}}\right)$$

The dynamic quantities required to regulate the blockchain are captured by the state ω. The control vector $C_{v}$ should be built to activate the blockchain.

(50) $$\omega =\left[T_{c}\;,S_{t}^{U_{d}}\;,S_{t}^{tk}\;,T_{t}^{cp}\right],\;\;\;\;\;\;\;\;\;C_{v}=\left[C_{t}^{p},\;T_{a},\;A_{s}^{po}\right]$$

PDPoS does not necessitate the costly and powerful equipment required to enable network functioning for IoT devices. As a result, network maintenance costs are reduced. Furthermore, PDPoS networks are environmentally friendly because they capitalize on little power.

The blockchain’s sustainability is critical, which is attained by utilizing PDPoS for the depletion of threats because it serves as a protection for the blockchain network. To do this, the smart contract managed by PDPoS dynamically distributes block rewards across blockchain-enabled IoT devices. This is accomplished by employing a time-varying parameter β(t) ranging between 0 and 1.

Thus, the transaction rate during the specific time $D_{r}\left(t\right)$ can be determined as follows:

(51) $$D_{r}\left(t\right)=U_{r}\left(t\right)\cdot \left(B_{r}\left(t\right)+C_{r}\cdot S_{y}\left(t\right)\cdot \beta \left(t\right)\right)$$

$$B_{r}\left(t\right)=FU_{r}\left(t\right)$$

(52) $$N_{br}\left(t\right)=C_{r}\cdot S_{y}\left(t\right)\cdot \left(1-\beta (t)\right)-B_{r}\left(t\right)$$

where $B_{r}\left(t\right)$ denotes the block-generating rate, $C_{r}$ is an average collateral ratio, S−y(t) represents the reward earnings, and *F* is a function that grows indefinitely.

The modification of the time-varying parameter β(t) becomes compatible with the block-generating rate $A_{r}\left(t\right)$ that is critical to the stable system. The PDPoS algorithm for β(t) works in discrete time steps, as shown below:

(53) $$\beta \left(t\right)+1=\Omega \left(\frac{A_{r}\left(t\right)}{D_{r}\left(t\right)}\right)\beta \left(t\right)$$

where Ω denotes a monotonically growing concave function

Assume for the time being that an IoT-enabled user on the blockchain is risk-neutral and does not devalue the performance. Since the process happened over a short period of time, discounting should not really be a factor. A useful and significant benchmark is provided by rationality and risk neutrality. Next, take into account a date 0≤t<T and a brief window of time (t,t+Δ) from *t* to *t*+Δ,Δ>0. We suppose that during this time, the communication among the IoT-enabled user $P_{t}$ remains roughly constant.

Let us assume that a number of transactions $T_{t}^{U}\Delta$ are initiated and converted into secured blocks over the edge computer for the IoT devices. Thus, the secured blocks can be determined as follows:

(54) $$L_{n}\Delta =L_{n}+\left(\frac{T_{t}^{U}}{P_{t}}\right)$$

where $L_{n}$ denotes the number of secured blocks.

PDPoS provides a fair voting process for selecting the delegates that request for transaction validation. The fair voting process is completed at time t+Δ with a higher success probability $\gamma_{t}\Delta$, and the success will be $P_{t}+\Delta =M_{c}^{t}\Delta +/L_{n}+\Delta$. The fair voting process continues with a probability of $1-\gamma_{t}\Delta$, and the success rate during the operating process is $P_{t}+\Delta$. On the other hand, it is also important to count the number of transactions for IoT devices $T_{t}^{U}/P_{t}$ and the number of blocks $M_{c}^{t}\Delta$ at time t+Δ, together with risk neutrality and rationality for the number of IoT-enabled users $P_{t}$. On the other hand, the order for the quantity of blocks for the number of IoT-enabled users $P_{t}$ can be determined as follows:

(55) $$P_{t}=\left(\frac{m_{t}^{v}+\Delta }{L_{n}\Delta }\times \gamma_{t}\Delta \right)+\left(1-\gamma_{t}\Delta \right)P_{t}\Delta$$

If the numbers of blocks at time *t* cannot match with the the transaction-generating rate at t+Δ, which can be determined by looking at the number of delegates in the blockchain technology, $m_{t}^{v}=M_{c}^{t}P_{t}$. At the start of the time span (t,t+Δ), the number of original blocks in the network is $m_{t}^{v}$. Now, it can be written as $m_{t}^{v}=m_{t}^{v}+T_{t}^{USD}$. IoT-enabled users are not willing to accept the sudden increase in blocks in the network. The process will finish at t+Δ with a success probability of $\gamma_{t}\Delta$, and the number of blocks will be $M_{c}^{t}\Delta$. The run continues with a success probability of $1-\gamma_{t}\Delta$, and the total blocks at the start of the subsequent time interval are $m_{t}^{v}+\Delta$.

(56) $$M_{c}^{t}+T_{t}^{U}=\left(\gamma_{t}*m_{t}^{v}\Delta \right)+\left(1-\gamma_{t}\Delta \right)M_{c}^{t}+\Delta$$

where $M_{c}^{t}\Delta$ denotes the network capacity.

Instability in the blockchain technology will undoubtedly affect the other; however, small variations can be tolerated. The instability in the blockchain can be modeled using stochastic differential equations, which explain how a network responds in uncertain settings. One such practical mathematical model is the scalar case, which can be expressed as a stochastic linear differential equation.

(57) $$\frac{dx(t)}{dt}=\beta \left(\zeta \left(t\right)x\left(t\right)\right),$$

(58) x(0)=ϕ(γ)

where coefficient (β) depends on the semi-Markov process (ζ(t)). The expected states ($\Theta_{1},\ldots ,\Theta_{n}$) of the stochastic process (ζ(t)) illustrate the situations in which the blockchain efficiently works, for instance, in the stable blockchain, during a cyberattacks, and so forth. Let circumstance ($\Theta_{k}$), k = 1, 2, …n, be taken by the stochastic process (ζ). Thus, it can be denoted that β(ζ(t)) = ($\beta_{k}$). The following equations can also be used to show the intensities ($\mu_{k}\left(t\right)$).

(59) $$\mu_{k}\left(t\right)=0$$

(60) $$\mu_{k}\left(t\right)=\left\{\begin{array}{c} \frac{1}{T_{jk}}for\;0\leq t<T_{jk}\\ \;0\;for\;t\geq T_{jk}, \end{array}\right.$$

Changes in the stochastic process (ζ(t)) are brought about by fluctuations in the network, and as a result, the solution in this scalar situation is subject to arbitrary transformations.

(61) $$x\left(t_{i}+0\right)=v_{k}^{x}\left(t_{i}-0\right),v_{k}\neq 0,k=1,\ldots ,n$$

At the time of the jumps ($t_{i}$), i = 1, 2, …

**Corollary 3.**  *It is possible to discover the potential threats in a specific situation.*

**Proof.**  The following three alternative states can be used to gauge the effectiveness of the blockchain technology employing the PDPoS algorithm. □

- If the blockchain is operating during the crisis, then β(ζ(t)) = ($\beta_{1}$);
- If the blockchain functions in a secure manner, then β(ζ(t)) = ($\beta_{2}$);
- If the blockchain works under restricted conditions, then β(ζ(t)) = ($\beta_{3}$).

With given concentrations, it can written as follows:

(62) $$\begin{array}{c} {\left(Z_{11}\right)}^{\left(t\right)}={\left(Z_{22}\right)}^{\left(t\right)}={\left(Z_{33}\right)}^{\left(t\right)}=0,{\left(Z_{12}\right)}^{\left(t\right)}={\left(Z_{13}\right)}^{\left(t\right)}={\left(Z_{21}\right)}^{\left(t\right)}={\left(Z_{23}\right)}^{\left(t\right)}=\\ {\left(Z_{31}\right)}^{\left(t\right)}={\left(Z_{32}\right)}^{\left(t\right)}\equiv \left\{\begin{array}{c} \frac{1}{T}\;for\;0\leq t<T,\\ \;0\;for\;t>T \end{array}\right.\;\;\;\;\;\;\;\;\;\;\;\;\; \end{array}$$

This demonstrates that with the use of PDPoS algorithm, the blockchain can remain stable for a certain amount of time ($\frac{1}{T}$).

The preceding three states demonstrate how blockchain technology can be used to effectively mitigate threats. The following alternative states can be modeled as follows:

(63) $$\begin{array}{c} \psi_{1}\left(V_{m}\right)=\psi F_{1}\left(V_{m}\right)+\left(V_{m}\right)\left(\beta_{12}\right)\left(\psi_{2}\right)\left(V_{m}\right)+V_{m})\left(\beta_{13}\right)\left(\psi_{3}\right)\left(V_{m}\right),\\ \psi_{2}\left(V_{m}\right)=\psi F_{2}\left(V_{m}\right)+\left(V_{m}\right)\left(\beta_{21}\right)\left(\psi_{1}\right)\left(V_{m}\right)+V_{m})\left(\beta_{23}\right)\left(\psi_{3}\right)\left(V_{m}\right),\\ \psi_{3}\left(V_{m}\right)=\psi F_{3}\left(V_{m}\right)+\left(V_{m}\right)\left(\beta_{31}\right)\left(\psi_{1}\right)\left(V_{m}\right)+V_{m})\left(\beta_{32}\right)\left(\psi_{2}\right)\left(V_{m}\right), \end{array}$$

where *F* denotes the capacity of the blockchain technology when employing the PDPoS consensus algorithm, ($V_{m}$) represents the standard function when the blockchain is being operated during entire time (*T*), and (ψ) shows the nature of the network, which is used for blockchain technology.

## 4. Experimental Setup and Results

We executed a number of scenarios to demonstrate the efficacy of the proposed VNFSDN approach for detecting and responding to security threats in 6G networks. A testbed with a 6G network emulator, numerous virtual network features, and a software-defined network controller was used. Additionally, blockchain technology was employed supported by the PDPoS consensus algorithm.

### 4.1. Experimental Setup

We used the OpenDaylight (ODL) scalable open platform to customize and automate networks of any complexity and scope. The DLL also provides compatibility for a variety of southbound protocols. When controlling network resources, it offers more flexibility and customization possibilities.

Three pre-built open-source VNFs were employed. Open vSwitch was the first VNF used, aiding in network traffic routing and administration. The second VNF was Bro, which provides intrusion detection and analysis capabilities. The cloud has VNF Bro installed. It is made up of two modules: a log database and a log correlator. The sliding window corresponding to the slow scan detection threshold and whose size is the specified time threshold is part of the log correlator module. The sliding window’s possible probes are all compared in this module based on the traits that distinguish the vertical, horizontal, and scattered scans. These traits include probes that originate from the same attacker and are directed at the same port or host, or in the case of decentralized scans, probes that originate from various attackers and are directed towards the same address or port. A scan is detected and an alarm is sent to all Bro VNFs running in the cloud if a group of probes with the same characteristics exceeds the probe threshold within the sliding window. With the use of this process, port scanning can be detected proactively because multiple user VMs underlying the VNFs may become aware of a prospective scan before it happens. Once a VNF firewall chain and a Bro VNF can develop an automatic prevention of the scan, proactive detection is especially helpful against horizontal scans. We then simulated the network environment. Mininet is an open-source software that allows the creation of virtual networks using a single Linux kernel and is commonly used for SDN research and testing. This enables the establishment of a virtual network. Mininet simulation has the capability to accept new applications. Thus, it can support our new introduced PDPoS consensus algorithm.

### 4.2. Experimental Scenarios

We simulated state-of-the-art scenarios to evaluate the performance of the proposed VNFSDN approach. The proposed approach was compared with (FSIDS) [11], DeepVulSeeker [12] (HADIDS) [26], and (DINF) [30] using the same scenarios.

- **Scenario 1:** A cyberattack is generated for IoT devices in the presence of SDN and a 6G network, and the network availability using the VNFSDN, VNFSDN with a firewall, and competing methods are measured.
- **Scenario 2:** Different network security measures are evaluated for DDoS attack mitigation and found that the VNFSDN approach is the most effective in improving the packet loss rate for IoT devices.
- **Scenario 3:** The impact of the VNFSDN approach is evaluated on traffic management in a 6G network by measuring the response to gradually increasing the number of IoT devices or user equipments (UEs) in the network.
- **Scenario 4:** A high-traffic 6G network is employed with a VNFSDN and PDPoS algorithm to manage the traffic flow for the IoT devices in the presence of potential attacks.
- **Scenario 5:** The threat detection rate of different security configurations is compared using a network without security, VNFSDN, VNFSDN with a firewall, IDS system, and a firewall.
- **Scenario 6:** The effectiveness of various network security solutions is evaluated for preventing data insertion, deletion, and alteration attacks by employing the PDPoS algorithm, VNFSDN and 6G technology and other competing methods (HADIDS, DINF, FSIDS, DeepVulSeeker approaches).

### 4.3. Testing Results

Based on the experimental results, the following parameters were analyzed employing the above scenarios to determine the effectiveness of the proposed and competing solutions.

- Network availability;
- Packet loss;
- Response time;
- Average traffic management time;
- Threat detection with traffic intensity;
- Undetected data;
- Malicious threat detection accuracy.

#### 4.3.1. Network Availability

**Figure 7:**
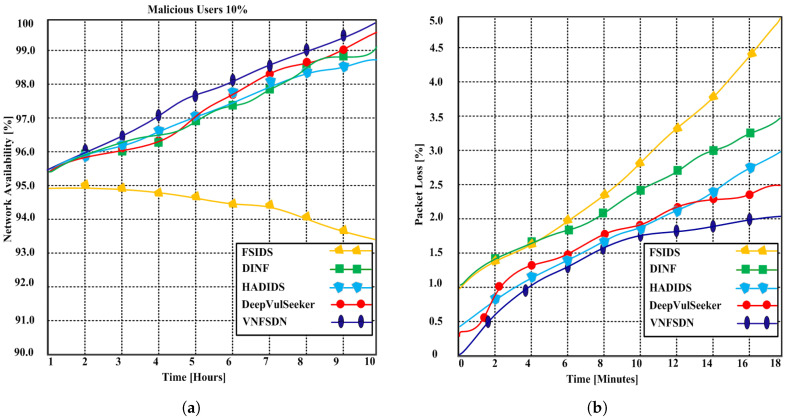
(**a**) Network availability with the proposed VNFSDN and competing methods. (**b**) Packet loss rate of the proposed VNFSDN and competing methods.

We simulated a distributed denial-of-service (DDoS) attack on the 6G network. An individual piece of user equipment on the network was the target of the attack, which creates a sizable amount of malicious traffic. The experiment used a network topology consisting of 10 hosts, a server, and a switch. We show the duration of network availability provided by the proposed VNFSDN and competing techniques.The proposed VNFSDN was the most successful at limiting the effects of the DDoS attack on the 6G network, as demonstrated by the results, which reveal 99.5% network availability during the 10 h testing period. A network availability of 99.63% was produced by the DeepVulSeeker, which is substantially greater than that of the other techniques, while other competing approaches HADIDS and DINF provided 90.01% and 98.72% network availability, respectively. The FSIDS approach was significantly affected, and network availability was decreased to 93.31%. The outcome shown in Figure 7a emphasizes how crucial security precautions are for preserving network availability. The topology with the VNFSDN offers the highest level of network availability and is the most secure option. On the other hand, FSIDS eventually faced more network breakdowns. The other approaches, such as DINF and HADIDS, provided intermediate levels of network availability.

#### 4.3.2. Packet Loss

To compute packet loss, many network security characteristics were analyzed for DDoS attack mitigation. Figure 7b depicts how the packet rate loss decreases as more security measures are implemented in the network. A total of 25,000 packets were transmitted over the network. We observed that the FSIDS produced the largest packet loss rate of roughly 1250, which is considered to be a packet loss of 5%, whereas the proposed VNFSDN had the lowest packet loss rates of 500, calculated to be a packet loss of 2%. The DeepVulSeeker droppred 625 packets, calculated as a packet loss of 2.5%. The topology with the VNFSDN also exhibited a considerable decrease in the packet loss rate when compared to the FSIDS approach, whereas the HADIDS and DINF revealed packet losses of 3.0% and 3.5%, respectively. It was concluded that the proposed approach has the capability to reduce the packet loss.

#### 4.3.3. Response Time

**Figure 8:**
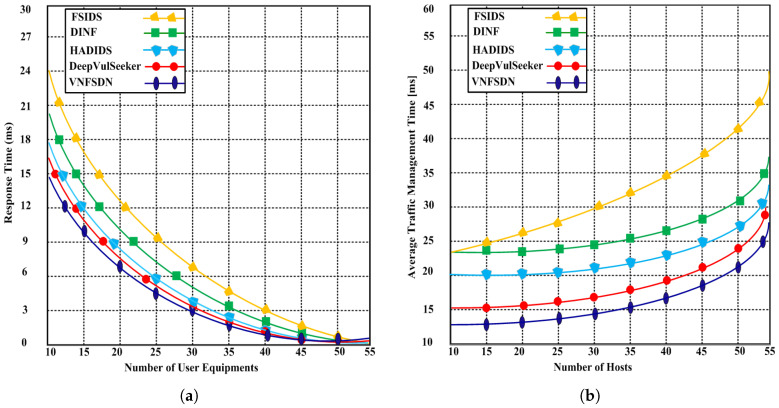
(**a**) Response times of the proposed VNFSDN and competing methods with a maximum of 55 UEs. (**b**) The average traffic management time of the proposed VNFSDN and competing methods with a maximum of 20 hosts.

The scalability of the VNFSDN and competing approaches were tested. The test was conducted by gradually increasing the number of user equipments in the network. The network topology consisted of a server, a switch, and a varying number of UEs ranging from 10 to 55. When the number of UEs increased, the response time greatly decreased. We measured the response time of the proposed VNFSDN and other competing approaches to detect and block malicious traffic in the network. Figure 8a depicts the response time for the proposed VNFSDN and competing approaches. The time taken for the server to reply to the query of a user is referred to as response time. The timer, which is expressed in milliseconds, begins when the user delivers a request and ends when the server that received the request returns the first response. The robust strategy requires less time to receive a response. Based on the results, we observed that the proposed VNFSDN approach shows a 0.08 ms response time with a maximum of 55 UEs. This demonstrates the robustness of the proposed approach. The primary reason for achieving a quicker response time in a shorter period was the use of 6G technology and the newly proposed PDPoS consensus method, which can prevent any malicious attack. As a result, the user receives a faster response with no intervention for packet loss. The competing DeepVulSeeker approach gave a 0.09 ms response time, but the other competing approaches, HADIDS and DINF, produced 0.28 ms and 0.328 ms response times, respectively. The FSIDFS yielded the highest response time 0.46 ms.

The response time $R_{t}$ can be calculated as follows:

(64) $$R_{t}=\sum_{i=1}^{U_{e}}i\left(T_{h}+T_{to}+Ttrn\right)$$

where $T_{h}$ denotes the time for the transaction to be processed by the host, $T_{to}$ signifies the time it takes for the transaction output to be transmitted from the host to the terminal through the network, $T_{rn}$ denotes the time at which the terminal must give a definite response to confirm receipt of the output, and $U_{e}$ denotes the total number of user equipments.

#### 4.3.4. Average Traffic Management Time

The practice of intercepting, examining, and allocating network traffic to the best resources in accordance with their priorities is known as network traffic management. Network performance, traffic statistics, and security are the main elements that need to be monitored for improved network management. A robust traffic management strategy includes load management and rate modeling, as well as QoS and availability of networks measures. To conduct the experiment, a network topology comprising a server, switches, and 55 hosts was used in the experiment. To manage network traffic flow, integration of SDN and VNF was deployed to balance the flow of traffic. The integration of multiple technologies increases the latency. To overcome this issue, Relation Degree (RD) was employed as proposed in [33]. RD describes the dependencies and interaction traffic in the service chain. Following the determination of the RD for each service chain pair, we deployed the function pairs on the same or nearby servers. This is a coordinated method that statically handles flows while taking traffic data into consideration during the placement step. It may reduce energy consumption and average transmission delay while managing network performance. The usage of 6G technology makes this a faster process to approach the network. Although SDN and VNF are dependent on one another, they can operate together to create network infrastructures that are adaptable and agile. Basic networking services are provided by the VNF, and higher-level management responsibilities are taken on by SDN to coordinate all network operations. Thus, the integration of both greatly helps in managing a faster network. The deployment of edge computing minimizes network latency by bringing storage, computing, and network capabilities closer to the application being used. Furthermore, the use of edge computing lowers the bandwidth, resulting in lower costs. There is a chance that private and sensitive traffic could be intercepted. As a result, the blockchain-enabled PDPoS consensus method was used to avoid malicious traffic.

We collected data for 10 min and estimated the average time for traffic management. The scenario used a time variable to track the threat detection rate over a predetermined length of time and an intensity level variable to imitate the quantity of network traffic. Figure 5b illustrates that the proposed VNFSDN takes an average traffic management time of 27.4 ms, while DeepVulSeeker took slightly longer, counted as 30 ms, and FSIDS took slightly longer, counted as 47.2 ms. The HADIDS required 33.01 ms of traffic management time, whereas the DINF required 37.6 ms.

#### 4.3.5. Threat Detection with Traffic Intensity

**Figure 9:**
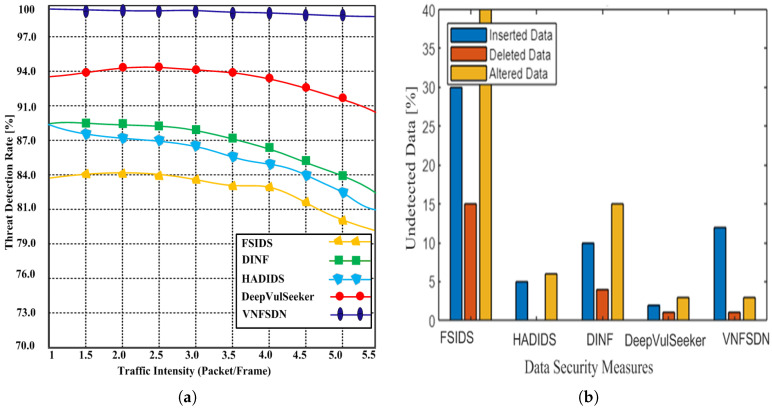
(**a**) The threat detection rate of the proposed VNFSDN and competing methods with a maximum traffic intensity of five (packet/frame). (**b**) The data integrity rate of the proposed VNFSDN and competing methods.

The threat detection rate of different security configurations on a network was determined. Figure 9a demonstrates that the proposed VNFSDN provides the highest threat detection rate among all the approaches, which was calculated at 99.36%. The DeepVulSeeker provided a 90.05% threat detection rate. On the other hand, the FSIDF approach showed a poor threat detection rate of 80.23%; this was because this approach does not have substantial support for security, whereas HADIDS showed an 81.0% detection rate, and DINF produced an 82.62% threat detection rate. The main reason for obtaining the highest detection rate for the proposed VNFSDN and DeepVulSeeker was to obtain the lowest false negative rate, which means that it is highly effective in detecting actual threats. The DeepVulSeeker approach had a lower threat detection rate compared to the VNFSDN, but it performed better than HADIDS, DINF, and FSIDS. The competing approaches, on the other hand, achieved malicious detection accuracy rates of 96.88%, 97.35%, 96.74%, and 97.62% for FSIDS, DINF, HADIDS, and DeepVulSeeker, respectively.

#### 4.3.6. Undetected Data

This experiment proved the accuracy of the data integrity. The capacity of several network security solutions to prevent three types of attacks, namely data insertion, data deletion, and data alteration, was determined. The VNFSDN was compared to HADIDS, DINF, FSIDS, and DeepVulSeeker. For each approach, we ran the scenario in a controlled environment with similar network configurations. Figure 9b demonstrates three different types of threats (data insertion, deletion, and alteration). The findings for data insertion reveal that the VNFSDN had the highest level of protection, with only 2% of inserted data going unnoticed. The HADIDS network was marginally less effective, with 5% of inserted data going undetected. The FSIDS provided the lowest level of protection, with 30% of inserted data going unnoticed. The HADIDS network provided the maximum level of data deletion protection, with no instances of deleted data going undetected. The VNFSDN was close behind, with only 1% of deleted data going unnoticed. The DeepVulSeeker and DINF networks offered nearly identical levels of security, with 3% and 4% of deleted data going undetected, respectively. The FSIDS provided the least protection, with 15% of deleted data escaping undetected. The VNFSDN had the highest level of security against data alteration, with only 3% of altered data remaining undetected. The HADIDS network was marginally less effective, with 6% of altered data remaining undetected. The DeepVulSeeker and DINF networks had 12% and 15% of altered data passing undetected, respectively. The FSIDS provided the least amount of security, with 40% of altered data passing undetected.

#### 4.3.7. Malicious Threat Detection Accuracy

**Figure 10:**
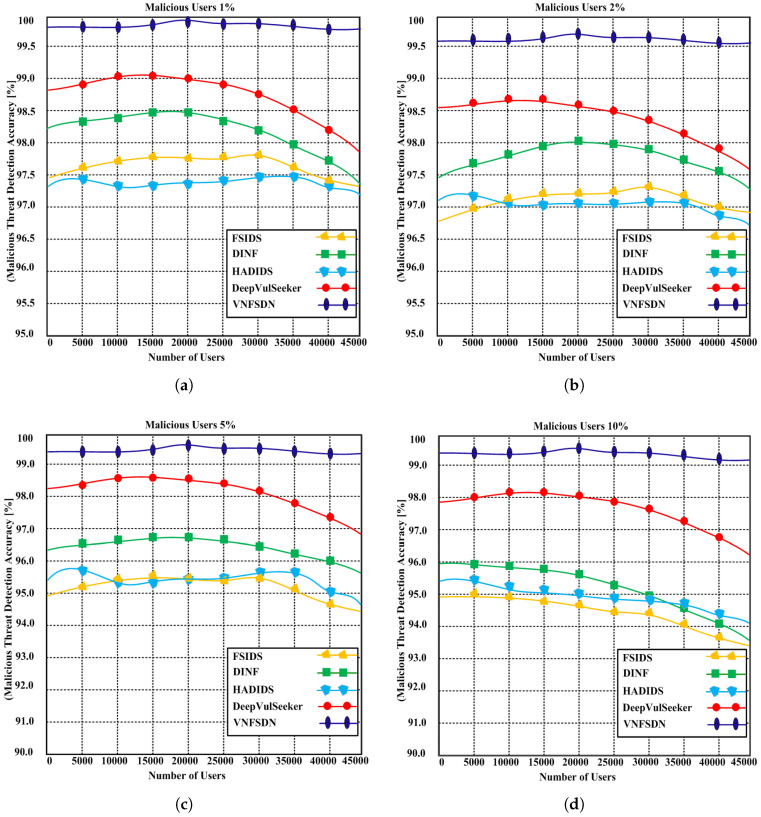
(**a**) Malicious threat detection accuracy of the proposed VNFSDN and competing methods with a maximum of 45,000 users and 1% malicious users. (**b**) Malicious threat detection accuracy of the proposed VNFSDN and competing methods with a maximum of 45,000 users and 2% malicious users. (**c**) Malicious threat detection accuracy of the proposed VNFSDN and competing methods with a maximum of 45,000 users and 5% malicious users. (**d**) Malicious threat detection accuracy of the proposed VNFSDN and competing methods with a maximum of 45,000 users and 10% malicious user.

Malicious detection is the identification of potentially harmful URLs and IP addresses that are known to be associated with threats and exploits such as malware, phishing, social engineering, and so on. When harmful URLs and internet protocols (IPs) are identified, they are utilized in security tools and apps to safeguard networks, endpoints, and users against domains, web pages, or IPs classed as malicious, phishing, deception, botnet, or one of the other types of exploits. Figure 10a depicts the malicious detection accuracy with a maximum of 45,000 users and 1% malicious users. The results show that the suggested VNFSDN technique achieved a greater detection rate of 99.77%, whereas the competing approaches achieved a lower detection rate of 97.45% for HADIDS, 97.41% for DINF, 97.32% for FSIDS, and 97.88% for DeepVulSeeker. For the FSIDS technique, a lower detection rate accuracy was computed. When the number of malicious users was increased to 2%, the proposed and competing approaches were not significantly different. Figure 10b demonstrates that with the same number of users (45,000), the suggested VNFSDN achieved 99.57% malicious detection accuracy. When the number of malicious users was increased to 5%, the proposed VNFSDN was not greatly affected; however, on the other hand, the competing approaches showed a reduced malicious reduction rate accuracy. Figure 10c shows the threat detection accuracy rate, and the proposed VNFSDN obtained a 99.36% threat detection accuracy, while the competing methods FSIDS, DINF, HADIDS and DeepVulSeeker produced threat detection rates of 94.49%, 95.78%, 94.71%, and 96.89% respectively. When the number of malicious users was increased to 10%, the proposed VNFSDN still delivered a consistent threat detection rate; however, the competing methods’ malicious detection rates were significantly. Figure 10d shows that the proposed VNFSDN had a 99.22% threat detection rate, while the competing methods FSIDS, DINF, HADIDS, and VNFSDN had rates of 93.41%, 93.61%, 94.19%, and 96.28%, respectively. The key reason for the VNFSDN’s increased malicious threat detection rate was the use of blockchain technology with the new PDPoS consensus algorithm, SDN and 6G technology.

## 5. Discussion

The findings indicate that the VNFSDN technique is effective in identifying and preventing DDoS attacks. The reaction time was reduced to 0.08 ms, demonstrating that the VNFSDN technique could respond to an attack quickly. The packet loss rate decreased from 5% to 2% during the attack, indicating that more packets were successfully sent. The throughput increased from 50 Mbps to 100 Mbps during the attack, indicating that more data could travel over the network. The VNFSDN strategy also increased network availability from 95% to 99.5%, indicating that the network was more reliable and accessible during the attack. The VNFSDN strategy successfully mitigated DDoS attacks and enhanced network availability and performance. The aforementioned statistics prove that the proposed solution is better at preventing attacks, such as DDoS, than a network without VNFSDN. The VNFSDN approach was the most effective at mitigating DDoS attacks, with a great improvement in response time and a reduction in the packet loss rate compared to the HADIDS, DINF, FSIDS, and DeepVulSeeker approaches. The DeepVulSeeker provided some level of protection; however, the solution was not as effective as the VNFSDN approach in mitigating the impact of DDoS attacks. The proposed VNFSDN had the best overall performance. The proposed VNFSDN approach demonstrated good scalability with a gradual increase in the number of user equipments (UEs) in the network. The approach showed minimal impact on the low packet loss rate as the number of UEs increased from 10 to 100. However, there was a slight increase in CPU and memory utilization as the number of UEs increased. The adoption of blockchain technology powered by the PDPoS consensus algorithm sacrifices CPU and memory. If we require security, we can compromise on the CPU and memory slightly. In addition, a test was carried out to validate the effect of the VNFSDN method on traffic management in the presence of IoT devices in a high-traffic 6G network.

The VNFSDN’s response time was discovered to be significantly lower than that of the other approaches, showing that it is more effective. Furthermore, a test was conducted to evaluate the performance of the VNFSDN approach in managing the traffic flow in a high-traffic 6G network. The implementation of the VNFSDN significantly improved the network performance. It was observed that the performance of the VNFSDN approach improved during the DDoS cyberattack on a 6G network. Network availability gradually decreased with time because of the attack, although the performance of the VNFSDN approach was not explicitly shown in the graph; this provides context for understanding the network’s overall behavior during a cyberattack simulation. The proposed VNFSDN approach had the highest threat detection rate of 99.5%. The DeepVulSeeker was second with a threat detection rate of 99.23%, followed by the HADIDS approach with a threat detection rate of 90.10%. The DINF approach had a threat detection rate of 84.76%, which is higher than that of the FSIDS (12.25%). These results demonstrate the importance of having a form of security in place to detect threats. The effectiveness of the different security solutions was analyzed via their ability to prevent data insertion, deletion, and alteration attacks in a controlled network environment with similar configurations for each scenario. The results show that the network with VNFSDN provided the highest level of protection against data insertion and alteration attacks, with only 2% and 3% of the inserted and altered data remaining undetected, respectively. The network with the HADIDS was most effective in preventing data deletion attacks, with no instances of missing data remaining undetected. The network with VNFSDN closely followed, with only 1% of the deleted data remaining undetected. The network without security exhibited the lowest level of protection against all three types of attacks, with a significantly higher percentage of data passing through undetected. These findings suggest that implementing VNFSDN can provide comprehensive protection against various DDoS attacks in a network environment. The findings also show that the VNFSDN technique can improve the network performance in various scenarios and environments. However, each scenario was conducted in specific contexts and environments, and thus the results may not be generalizable to all situations. Additionally, the VNFSDN strategy may not be the optimal solution for every network security and performance issue, as other considerations such as cost, scalability, and ease of deployment must be considered. It can be concluded that the results suggest that the VNFSDN approach could be a viable solution for defending against cyberattacks, but further optimizations may be necessary to improve its efficiency. Table 2 and Table 3 demonstrates the comparative performance of the proposed VNFSDN and competing approaches.

## 6. Conclusions and Future Work

This section discusses the findings of the proposed approach and the future directions.

### 6.1. Conclusions

In this study, we proposed virtual network function virtualization with software-defined networking for efficient IoT cyberattack depletion. The proposed method was supported with 6G technology to ensure faster communication and security improvement. The proposed VNFSDN was employed on edge computing. The blockchain technology with the novel PDPoS consensus algorithm was integrated to provide flexibility, scalability and security. Several tests were performed to assess the efficiency of the proposed method in identifying and responding to security concerns. Our findings demonstrate that the VNFSDN technique is a practical way to increase the scalability and efficiency of network security while lowering the danger of cyberattacks. A DDoS attack was generated against a 6G network and the blockchain-enabled PDPoS consensus algorithm. The proposed VNFSDN with integrated support from other features decreased the response time and packet loss rate. The usefulness of the VNFSDN technique in increasing network resiliency and decreasing downtime after a cyberattack was also demonstrated by increased network availability compared to other competing approaches (HADIDS, DINF, FSIDS, and DeepVulSeeker). The VNFSDN approach was the most effective at mitigating DDoS attacks and improving network performance compared to other security solutions tested in the same scenarios. The VNFSDN approach provided a better response time, packet loss rate, and network availability than the other approaches. Finally, data integrity of the proposed VNFSDN and competing approaches were evaluated using three state-of-art threats (i.e., insertion, deletion and alteration). Two innovative state-of-the-art algorithms, named threat filtration and threat capture, were devised and implemented to appropriately filter potential threats from traffic and then capture those potential threats. Furthermore, both algorithms had O (log n) time complexity, making the threat detection procedure substantially faster. Overall, the proposed VNFSDN approach highlighted the importance of implementing security measures in the network to protect against cyberattacks and improve network performance. Overall, our research indicates that the VNFSDN strategy is a workable solution and efficient for cyberattack depletion.

### 6.2. Future Work

Future studies could investigate how the VNFSDN method performs in more complicated network environments as well as the efficacy of combining additional VNFs and SDN controllers. This could provide insights into how the method performs in diverse scenarios, enabling more efficient and effective implementation. Furthermore, the emphasis will be on SDN to determine the efficacy of a particular technology in complex environments. We will generate different types of attacks to check the validity of the proposed approach. Finally, additional research should examine the potential trade-offs associated with the higher CPU and memory consumption of the VNFSDN strategy when combined with other security solutions. This would help in developing technologies that could improve network performance while retaining adequate security safeguards. Additionally, the VNFSDN strategy may not be the optimal solution for every network security and performance issue, as other considerations such as cost and ease of deployment should also be considered in the future.

## Figures and Tables

**Table 1:** Contemporary related works and their limitations.

Related Works	Solutions	Characteristics	Limitations
Rani et al. [20]	Machine learning-basedintrusion detection system forsoftware-defined networking	Predicts network traffic andoptimizes network performance	Requires a large amountof data to train the algorithms
Ahmed et al. [21]	A survey on networkvirtualization techniques andchallenges	Provides a comprehensive overview ofnetwork virtualization techniques andtheir potential benefits inimproving network performance,flexibility, and management	The proposed solutions maynot be applicable to allnetwork virtualization scenarios
Wang and Zhao [22]	A survey of mobile edgeComputing for the Metaverse:architectures, applications, and challenges	Reduces latency and bandwidthrequirements and improves networkperformance	Limited processing power andstorage capacity at thenetwork edge
Karakus and Durresi [23]	Quality of service (QoS)in software-defined networkingsurvey	Prioritizes network traffic andallocates bandwidth more effectively	Complexity in designing andimplementing QoS policies
Li et al. [24]	BlockCSDN:blockchain-based collaborativeintrusion detection insoftware-defined networking	Decentralized and tamper-proofrecord of network activity andimproved network security	High computational overheadand scalability issues
Yang et al. [25]	Advances in resource allocationin network function virtualization	Generalized and examined fourtypical resource allocation issuesfor QoS improvement and delaycalculation	The proposed solutions maynot be applicable to allNFV scenarios
Xu et al. [26]	Hybrid cloud computing:state-of-the-art, challenges,and future directions	Improves the security, privacy,and performance of hybridcloud systems	Requires additional resources andexpertise to implement andmaintain
Basu et al. [27]	QoS-aware dynamic networkslicing and VNF embeddingin softwarized 5G networks	Offers significant benefits interms of energy-efficientservice delivery, low latency,and optimized network efficiency	The MILP-based optimizationapproach may pose computationalchallenges in larger networkscenarios, and the approachmay require further validationin real-world deployments
Kim and Kim [28]	VNF placement methodbased on VNF Characteristics	Efficient VNF placement usinginformation about the resources of eachnode, which can lead to improvednetwork performance and resourceutilization	Increased complexity in termsof resource monitoring andupdating, as well as potentialscalability issues if thenumber of nodes and VNFsincrease significantly
Taniguchfi and Shinomiya [29]	A method of service functionchain configuration to minimizecomputing and network resourcesfor VNF failures	Reduces the computing resourcesrequired for SFC configurationcompared to previous studies,which can lead to cost savingsfor network operators	The proposed method maynot be suitable for alltypes of virtualized networks,and further research isneeded to evaluate itseffectiveness in different contexts
Yao et al. [30]	Anomaly-based approach forIoT	This approach improves the detectionaccuracy and reduces false positives.The main advantage of this approachis its ability to detect both knownand unknown attacks	Potential for higher resourceconsumption due to running multipledetection methods simultaneously
Zheng et al. [31]	Network firewall for cybersecurity in an IoT environment	The approach improves securityand reduces the potential for falsepositives. The main advantage of thisapproach is its ability to adaptto changing network conditions	The potential for higher resourceconsumption due to continuousanalysis and rule updates
Our Work	VNFSDN	Improved network performance andscalability and increased networkefficiency	Increased complexity andmanagement overhead due to theneed for specialized skills andtools to manage the virtualizednetwork functions

**Table 2:** Comparative analysis of the proposed VNFSDN and competing approaches based on the findings.

Approaches	Response Time	Packet Loss Rate	Network Availability	Traffic Management Time	Threat Detection Rate
(FSIDS) [11]	0.46 (ms)	5.0%	93.31%	47.2%	80.23%
DeepVulSeeker [12]	0.09 (ms)	2.5%	99.63%	30.0%	90.05%
(HADIDS) [26]	0.28 (ms)	3.0%	99.01%	33.01%	81.0%
(DINF) [30]	0.328 (ms)	3.5%	98.72%	37.6%	82.62%
**Proposed VNFSDN**	0.08 (ms)	2.0%	99.5%	27.4%	99.36%

**Table 3:** Comparative analysis of the malicious threat detection accuracy for the proposed VNFSDN and competing methods with a maximum of 45,000 users and 1%, 2%, 5%, and 10% malicious users.

Approaches	MD Accuracy 1%	MD Accuracy 2%	MD Accuracy 5%	MD Accuracy 10%
(FSIDS) [11]	97.32%	96.88%	94.49%	93.41%
DeepVulSeeker [12]	97.88%	97.62%	96.89%	96.28%
(HADIDS) [26]	97.45%	96.74%	94.71%	94.19%
(DINF) [30]	97.45%	96.74%	95.78%	93.61%
**Proposed VNFSDN**	99.77%	99.57%	99.36%	99.22%

## Data Availability

Data are contained within the article.
